# Coelacanth *SERINC2* Inhibits HIV-1 Infectivity and Is Counteracted by Envelope Glycoprotein from Foamy Virus

**DOI:** 10.1128/JVI.00229-21

**Published:** 2021-06-10

**Authors:** Pavitra Ramdas, Vipin Bhardwaj, Aman Singh, Nagarjun Vijay, Ajit Chande

**Affiliations:** a Molecular Virology Laboratory, Department of Biological Sciences, Indian Institute of Science Education and Research, Bhopal, India; b Computational Evolutionary Genomics Lab, Department of Biological Sciences, Indian Institute of Science Education and Research, Bhopal, India; Ulm University Medical Center

**Keywords:** SERINC, restriction factors, antiretroviral, whole-genome duplication, foamy viruses

## Abstract

*SERINC5* restricts nef-defective HIV-1 by affecting early steps of the virus life cycle. Distantly related retroviruses with a wide host range encode virulent factors in response to challenge by *SERINC5*. However, the evolutionary origins of this antiretroviral activity, its prevalence among the paralogs, and its ability to target retroviruses remain understudied. In agreement with previous studies, we found that four human *SERINC* paralogs inhibit nef*-*defective HIV-1, with *SERINC*2 being an exception. Here, we demonstrate that this lack of activity in human *SERINC*2 is associated with its post-whole-genome duplication (post-WGD) divergence, as evidenced by the ability of pre-WGD orthologs from Saccharomyces cerevisiae and flies and a post-WGD-proximate *SERINC*2 from coelacanths to inhibit the virus. Intriguingly, Nef is unable to counter coelacanth *SERINC*2, indicating that such activity was directed toward other retroviruses found in coelacanths (like foamy viruses). However, foamy virus-derived vectors are intrinsically resistant to the action of *SERINC*2, and we show that the foamy virus envelope confers this resistance by affecting its steady-state levels. Our study highlights an ancient origin of antiretroviral activity in *SERINC*s and a hitherto-unknown interaction with a foamy virus.

**IMPORTANCE** *SERINC*5 constitutes a critical barrier to the propagation of retroviruses, as highlighted by parallel emergence of anti-SERINC5 activities among distant retroviral lineages. Therefore, understanding the origin and evolution of these host factors will provide key information about virus-host relationships that can be exploited for future drug development. Here, we show that *SERINC5*-mediated nef-defective HIV-1 infection inhibition is evolutionarily conserved. *SERINC*2 from coelacanth restricts HIV-1, and it was functionally adapted to target foamy viruses. Our findings provide insights into the evolutionary origin of antiretroviral activity in the *SERINC* gene family and uncover the role of SERINCs in shaping the long-term conflicts between retroviruses and their hosts.

## INTRODUCTION

Retroviruses exploit a wide host-range for their persistence, in response to which host species have continually evolved increasingly intricate antiviral defense strategies (1). As part of this ongoing arms race, while viruses have relied on the acquisition or fusion of diverse genes, the host defense mechanisms have been greatly strengthened by the functional divergence of gene copies following duplication of genes as well as whole genomes (2–6). Not just the host defense mechanism but the overall success of the vertebrate lineage is thought to have benefited immensely from the two whole-genome duplication events which have occurred over the course of evolution (2). While the study of gene and genome duplication has undoubtedly improved our understanding of how evolution works, it has also become a useful approach for obtaining important insights about the functional intricacies of restriction factors (4–7). Tracking the evolutionary history of restriction factors based on their origin, loss, duplication, and increased rates of sequence evolution concomitant with the change in the pathogen repertoire has been used as a strategy to map the diversification of various restriction factors (8–10). The most prominent signature of this ongoing arms race is seen in the form of a strong, recurrent positive selection at functionally important residues of the restriction factor genes (11, 12). Restriction factors, being at the forefront of long-term host-virus conflict (13–15), show clear molecular signatures indicative of an arms race (11, 12). In fact, the presence of these signatures has been proposed as a hallmark of restriction factors (14, 16) and has been employed as a screening strategy to identify putative candidates with antiviral activities (4, 17).

In contrast, the antiretroviral host factors *SERINC*5 and *SERINC*3 display a comparatively uneventful evolutionary history (18, 19). This is counterintuitive, because distant retroviruses with a wide host range encode anti-*SERINC*5 virulent factors in response to challenge by *SERINC5* (20–23). Hence, we sought to trace the evolutionary origins of the antiretroviral activity of *SERINC*5, its prevalence among the *SERINC* paralogs, and its relevance for retroviral inhibition. Our analysis to comprehend the evolutionary origins of the antiretroviral function of *SERINC*s identified an antiretroviral *SERINC*2 with a hitherto-unknown interaction with a foamy virus.

## RESULTS

### Antiretroviral activity among human *SERINC* paralogs and evolutionary trajectories.

**FIG 1:**
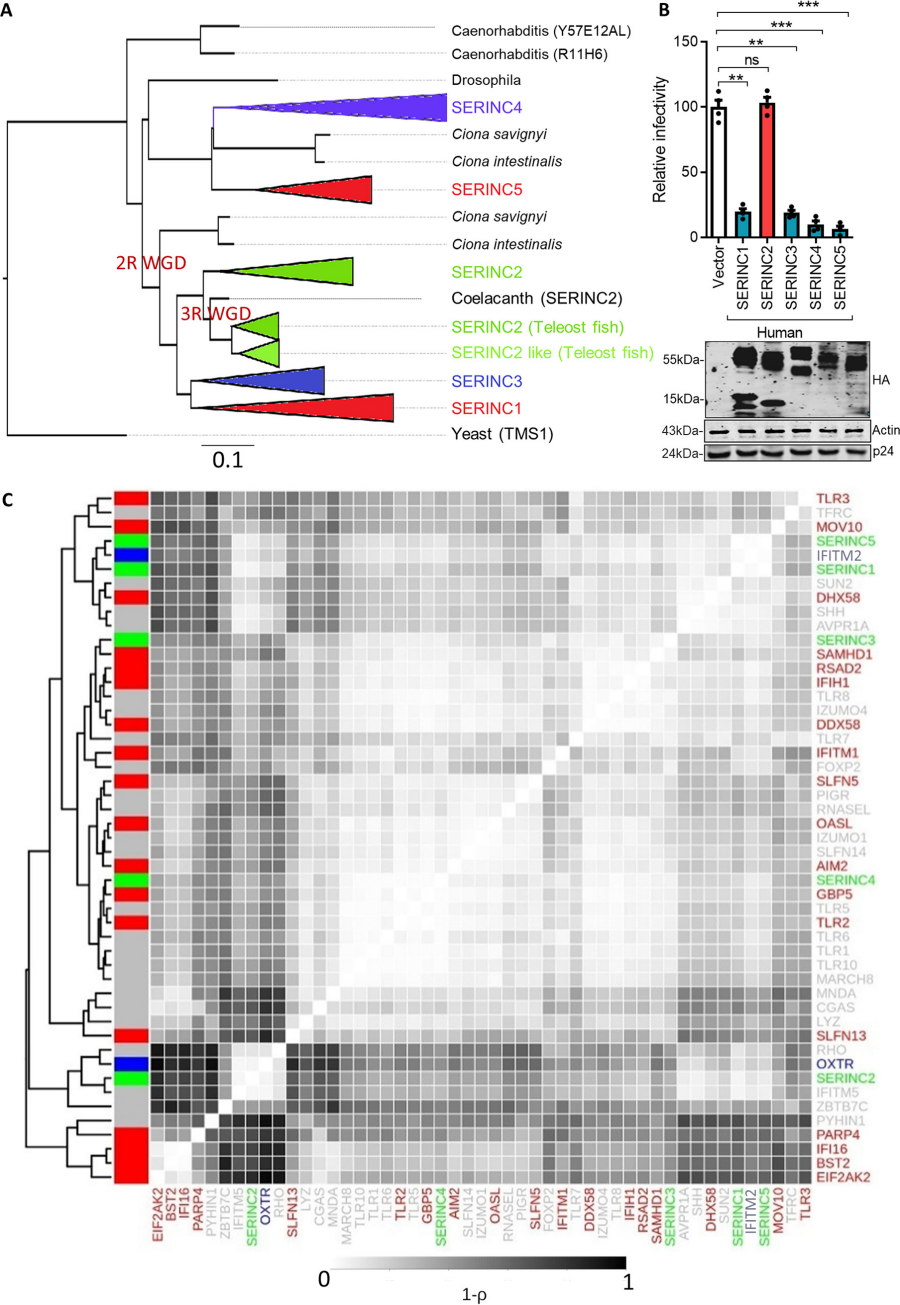
The activity of human *SERINC* paralogs on HIV-1 infectivity and evolutionary history and arms race signatures of *SERINC* genes. (A) Phylogenetic analysis of *SERINC* paralogs. S. cerevisiae, Caenorhabditis elegans, and D. melanogaster have a single copy of this gene. Whole-genome duplication that occurred in an ancestor of mammals gave rise to multiple *SERINC* genes. The *SERINC* genes form two clusters consisting of *SERINC*1, *SERINC*2, and *SERINC*3 and of *SERINC*4 and *SERINC*5. The members of the first cluster, *SERINC*1, -2, and -3, are grouped as ohnologs based on a strict Q-score criterion by the http://ohnologs.curie.fr/ database. The second cluster, consisting of *SERINC*4 and -5, potentially reflects a subsequent duplication event that is shared by most of the post-WGD species. Colors denote different branches of each *SERINC* in chordates. The lengths of the triangles are proportional to the number of nucleotide substitutions that have taken place in a particular branch. The tree was generated using Ensembl. (B) (Top) A single-cycle infectivity assay was performed using TZM-GFP cells for nef-defective HIV-1 particles that were produced from HEK293T cells by transfecting 100 ng of indicated human SERINC paralogs encoded by pcDNA3.1(−), SERINC5 encoded by PBJ6, or the empty vector backbone along with pHXB2 envelope and NL4-3 Env(−) Nef(−). The infectivity was normalized to RT units using an SGPERT assay. The values obtained from the empty vector control were set to 100 for comparison with SERINC expressors. Values are means (*n* = 4) and standard deviations (SD). An unpaired *t* test was used to determine significance. **, *P* < 0.01; ***, *P* < 0.001; ns, not significant. (Bottom) Western blot showing expression of C-terminally HA-tagged *SERINC* and the corresponding β-actin and HIV-1 p24 from HEK293T cell lysate. (C) Hierarchical clustering of the arms race signatures in primate genes aligned using ClustalW aligner and used as input for FUBAR. Interferon-induced genes identified in reference 30 are color coded red (upregulated), blue (downregulated), green (*SERINC* paralogs), and gray (other selected genes). FUBAR quantifies evolutionary fingerprints of a gene by dividing the synonymous (α) and nonsynonymous (β) rates into a finely discretized grid. Each point in this grid represents a pair of α and β values, with the size of the circle representing the value θ̂ (the posterior mean), which provides a measure of how many of the sites in the multiple-sequence alignment contribute to that particular combination of α and β values. The vector of 400 θ̂ values that correspond to each of the points on the 20-by-20 grid of α and β values serves as the evolutionary fingerprint of a gene. Genes with canonical arms race signatures have a greater weight for grids with higher values of β than genes that lack the arms race signature (19).

Analysis of sequence similarity and gene structure conservation reveals that *SERINC*5 and *SERINC*4 share a recent ancestry (Fig. 1A). Similarly, *SERINC*3 and *SERINC*1 paralogs are most similar to each other (∼60% identity). Despite having the lowest sequence similarity to either of the established antiviral *SERINC* paralogs, *SERINC*2 is relatively similar to *SERINC*3 (50%) and *SERINC*5 (37%). Given such levels of sequence similarity and conserved membrane topology, we evaluated the ability of other *SERINC* paralogs to inhibit nef-defective-HIV-1 infectivity. We transiently transfected HEK293T cells with the individual *SERINC*s along with nef-defective HIV-1 (NL4-3 isolate). The viruses were collected, and infectivity assays were performed as reported previously (20, 21). In agreement with earlier reports (24–26), we confirmed that the inhibition of nef-defective-HIV-1 infectivity is conserved among four human *SERINCs*; *SERINC*2 is the only paralog that shows no detectable activity against the virus (Fig. 1B).

Based on the ability of human *SERINC*3 and *SERINC*5 to restrict nef-defective HIV-1, they were proposed as restriction factors (21, 22). However, in contrast to other restriction factors, *SERINC*5 and *SERINC*3 genes have a rather uneventful history that is distinct from the traditional signatures of recurrent selection seen in genes that are part of an arms race (19). We investigated whether the absence of signatures indicative of positive selection was prevalent across all the *SERINC* paralogs as a general feature. To this end, we compared the evolutionary signatures of *SERINC* genes with those of previously identified restriction factors—mainly genes showing recurrent positive selection and a few functionally characterized genes which act as controls (Fig. 1C). Although the arms race signatures of a few restriction factors such as BST-2 and EIAF2AK2 were well correlated, the other newly identified restriction factors, including *SERINC*s, did not show any consistent pattern of clustering (Fig. 1C). The *SERINC*5 transcript is not upregulated upon interferon (IFN) treatment (27, 28). However, one of the prime features of restriction factors is their ability to be augmented upon interferon stimulation, and this feature has been linked to the arms race with viruses (29). While the genes which formed a cluster are indeed interferon responsive (IFN-responsive genes were obtained from reference 30), this property does not explain the overall pattern of clustering (Fig. 1C). Furthermore, arms race signatures are not prominent in several interferon-inducible genes and innate immune genes, including Toll-like receptors (TLRs) (Fig. 1C). Therefore, the lack of arms race signatures in *SERINC*s is probably not very surprising. The ability to restrict HIV-1 among human SERINC paralogs suggests that this feature has been evolutionarily conserved despite their early divergence. This observation prompted us further to investigate the evolutionary origins of this antiretroviral activity.

### Anti-HIV-1 activity is an ancient feature among *SERINC*5 orthologs.

**FIG 2:**
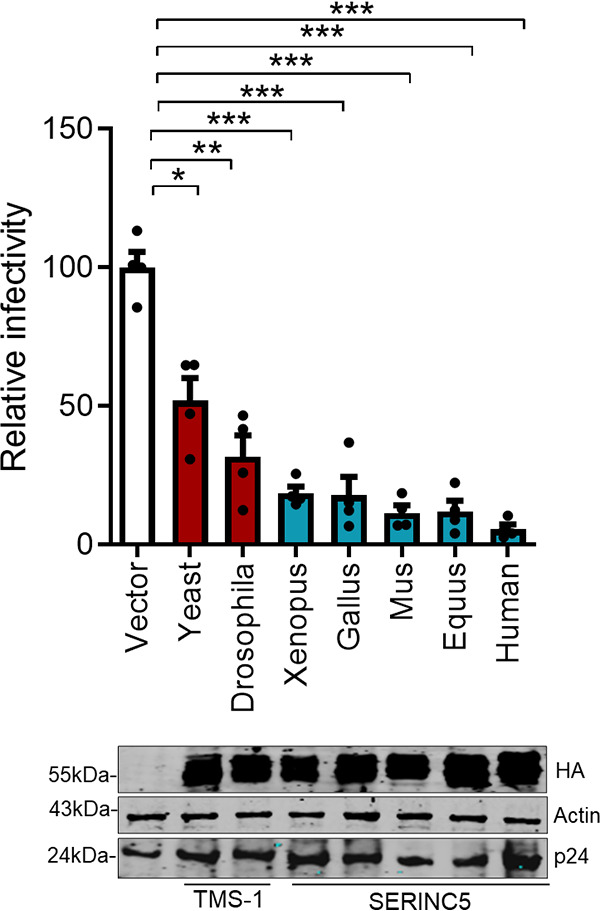
Activity of *SERINC5* orthologs on HIV-1 infectivity. (Top) A single-cycle infectivity assay was performed using TZM-GFP target cells for nef-defective HIV-1 particles produced from HEK293T cells by transfecting 100 ng of indicated pre- and post-WGD SERINC5 orthologs encoded by pcDNA3.1(−), human SERINC5 encoded by PBJ6, or the empty vector backbone along with pHXB2 envelope and NL4-3 Env(−) Nef(−). The infectivity was normalized to RT units using SGPERT assay. The values obtained from the empty vector control were set to 100 for comparison with SERINC expressors. Values are means (*n* = 4) and SD. An unpaired *t* test was used. *, *P* < 0.05; **, *P* < 0.01; ***, *P* < 0.001. (Bottom) Western blot showing expression of C-terminally HA-tagged *SERINC5* orthologs and the corresponding β-actin and HIV-1 p24 from cell lysates (HEK293T).

*SERINC*s plausibly shaped retroviral evolution, as indicated by the parallel emergence of anti-*SERINC*5 activity among diverse retroviral genomes (18, 23, 31, 32). Since the *SERINC* gene family is conserved across eukaryotic species, we investigated the extent to which the ability of *SERINC* orthologs to restrict HIV-1 is conserved. Unicellular eukaryotes and invertebrates have a single copy of the *SERINC* gene (*TMS*1); all these orthologs could restrict nef-defective HIV-1 (Fig. 2). However, the yeast (Saccharomyces cerevisiae) ortholog showed a relatively modest activity (2-fold) in comparison to all the other orthologs (inhibition between 4-fold and 36-fold). All these experiments were conducted under comparable transfection conditions. We also confirmed the expression of C-terminally hemagglutinin (HA)-tagged TMS-1 and other *SERINC*5 orthologs by Western blotting from cell lysates (Fig. 2, lower panel). Overall, these results indicate that anti-HIV activity is an ancient and evolutionarily conserved feature among *SERINC*s that predates the whole-genome duplication event between ∼700 to 400 million years ago (MYA) (33).

### Coelacanth *SERINC*2 inhibits HIV-1.

**FIG 3:**
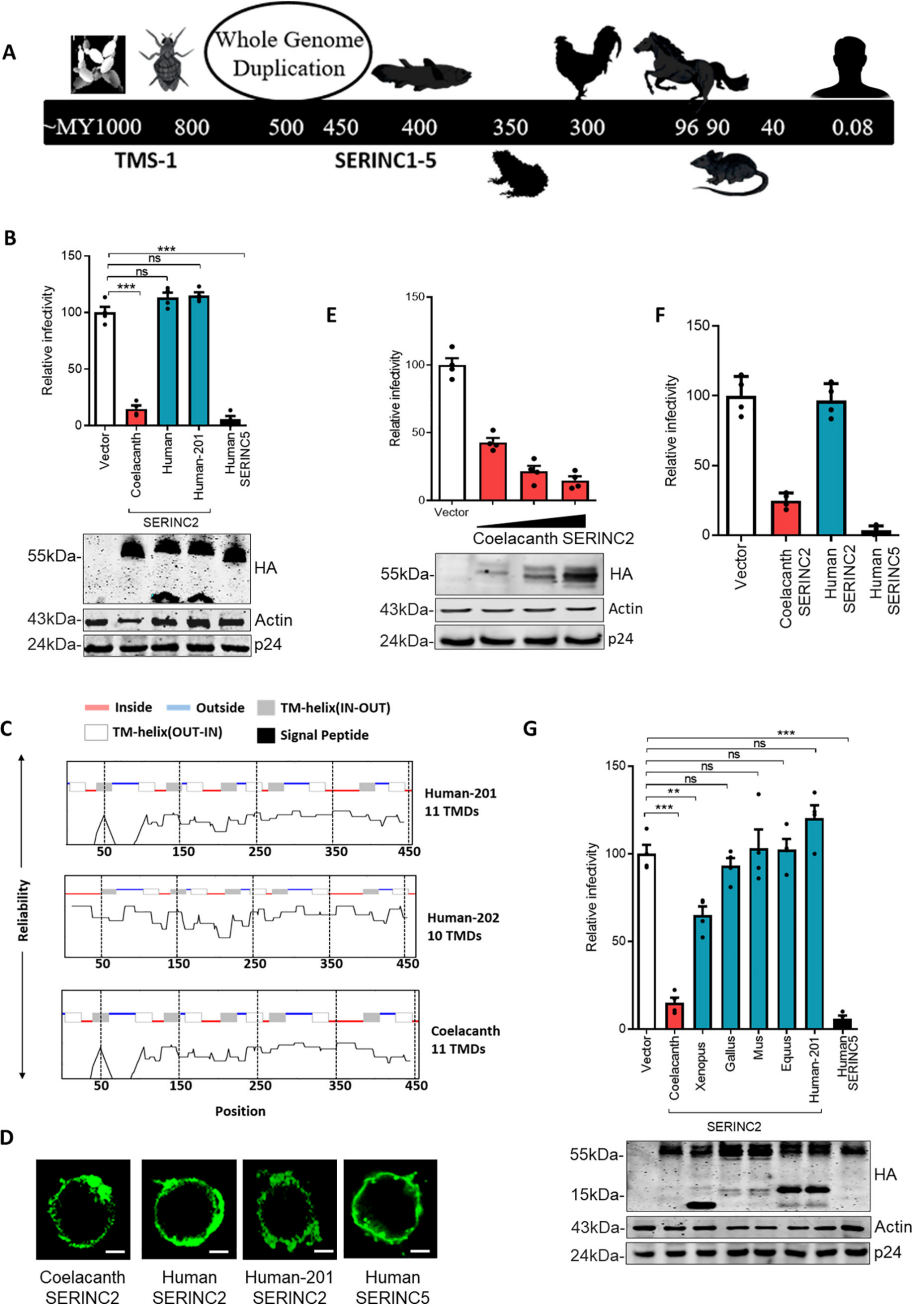
Coelacanth *SERINC*2 inhibits HIV-1 infectivity. (A) Schematic timeline depicting the sequence of events during the course of evolution, with the species (left to right) S. cerevisiae, D. melanogaster, *L. chalumnae*, *X. tropicalis*, *G. gallus*, *E. caballus*, *M. musculus*, and H. sapiens. (B) (Top) A single-cycle infectivity assay was performed using TZM-GFP target cells for nef-defective HIV-1 particles that were produced from HEK293T cells by transfecting 100 ng of coelacanth SERINC2, human SERINC2 isoforms encoded by pcDNA3.1(−), human SERINC5 encoded by PBJ6, or the empty vector backbone along with pHXB2 envelope and NL4-3 Env(−) Nef(−). The infectivity was normalized to RT units using an SGPERT assay. The values obtained from the empty vector control were set to 100 for comparison with SERINC expressors. Values are means (*n* = 4) and SD. An unpaired *t* test was used. ***, *P* < 0.001; ns, not significant. (Bottom) Western blot showing expression of C-terminally HA-tagged *SERINC* expressors and the corresponding β-actin and HIV-1 P24 from cell lysate (HEK293T). (C) Topological features of human *SERINC*2 splice isoforms and coelacanth *SERINC*2 predicted using TOPCONS (80). (D) Immunofluorescence of HA-tagged coelacanth SERINC2, human SERINC2 isoforms, and human SERINC5 transfected in JTAg*SERINC*5/3^−/−^ cells and visualized using mouse anti-HA primary antibody and mouse Alexa Fluor 488-conjugated secondary antibody. Bar, 10 μm. (E) (Top) A single-cycle infectivity assay was performed using TZM-GFP target cells for nef-defective HIV-1 particles that were produced from HEK293T cells, by transfecting coelacanth encoded by PBJ6, PBJ5, pcDNA3.1(−), or the empty vector backbone along with pHXB2 envelope and NL4-3 Env(−) Nef(−). The infectivity was normalized to RT units using an SGPERT assay. The values obtained from the empty vector control were set to 100 for comparison with SERINC expressors. Values are means (*n* = 4) and SD. (Bottom) Western blot showing expression of C-terminally HA-tagged coelacanth SERINC2 and the corresponding β-actin and p24 from cell lysates (HEK293T). (F) A single-cycle infectivity assay was performed using TZM-GFP target cells for nef-defective HIV-1 particles that were produced from JTAg *SERINC*5/3^−/−^, by transfecting coelacanth SERINC2, human SERINC2, and human SERINC5 encoded by pcDNA3.1(−) or the empty vector backbone along with pHXB2 envelope and NL4-3 Env(−) Nef(−). The infectivity was normalized to RT units using an SGPERT assay. The values obtained from the empty vector control were set to 100 for comparison with SERINC expressors. Values are means (*n* = 3) and SD. (G) (Top) A single-cycle infectivity assay was performed using TZM-GFP target cells for nef-defective HIV-1 particles that were produced from HEK293T cells by transfecting 100 ng of SERINC2 orthologs (coelacanth, *Xenopus*, *Gallus*, *Mus*, *Equus*, and human) encoded by pcDNA3.1(−) and human SERINC5 encoded by PBJ6 or the empty vector backbone along with pHXB2 envelope and NL4-3 Env(−) Nef(−). The infectivity was normalized to RT units using an SGPERT assay. The values obtained from the empty vector control were set to 100 for comparison with SERINC expressors. Values are means (*n* = 4) and SD. An unpaired *t* test was used. **, *P* < 0.01; ***, *P* < 0.001; ns, not significant. (Bottom) Western blot showing expression of C-terminally HA-tagged *SERINC* expressors and the corresponding β-actin and HIV-1 p24 from cell lysate (HEK293T).

Two rounds of whole-genome duplication (WGD) in the ancestor of chordates led to a substantial increase in the number of genes, thereby leading to the acquisition of new functions. This repertoire of genes also provides greater flexibility due to their mutually compensatory functions. Hence, post-WGD paralogous copies tend to diversify. A single copy of a *SERINC* ortholog (*TMS*1) is present in pre-WGD species, and post-WGD, the number of copies has increased to five in tetrapods and six in bony fishes (Fig. 1A). Our investigation of the pre-WGD ortholog of *SERINC* genes from yeasts (Saccharomyces cerevisiae) and flies (Drosophila melanogaster) *TMS1* found evidence of antiretroviral activity (Fig. 2). Functional data on the human paralogs that diverged early also suggest an ancestrally conserved activity of *SERINC*s in restricting HIV-1, with *SERINC*2 being the exception (Fig. 1B). Hence, the human *SERINC2* paralog may have lost the ability to restrict retrovirus sometime after the WGD event (∼700 to 400 MYA) (33). It is plausible that the species proximal to the WGD event might retain a version of *SERINC2* with antiviral activity (Fig. 3A). Therefore, we decided to systematically screen *SERINC2* orthologs from post-WGD species at various levels of sequence divergence from human *SERINC2*. To this end, we synthesized *SERINC*2 from various post-WGD species. The codons had to be optimized using the GeneArt portal for expression in human cells owing to their nonhuman origin. During virus production, coexpression of select *SERINC*2 orthologs and subsequent infectivity analysis revealed that coelacanth *SERINC*2 restricts nef-defective HIV-1 (∼7-fold) under conditions in which *SERINC*2 isoforms from humans did not show any activity (Fig. 3B). A shorter isoform of human *SERINC*2 (Human-201) was found to be topologically similar (Fig. 3C) to that of coelacanth *SERINC*2 but lacked the activity to restrict nef-defective HIV-1 concurrent with the longer isoform (Fig. 3B). The cellular localization of coelacanth SERINC2 was assessed and found to be comparable to that of SERINC2 isoforms and SERINC5 from human (Fig. 3D). We saw a dose-dependent inhibition when coelacanth *SERINC*2 was expressed from plasmids carrying promoters of various strengths (PBJ6-, PBJ5-, and pcDNA-based expression vectors; the expression levels are indicated in the Western blots), suggesting that this is not an artifact of overexpression (Fig. 3E). We also verified the activity of coelacanth *SERINC*2 from a T-cell line that lacked endogenous *SERINC*3 and *SERINC*5 using the same constructs used in HEK293T cells and found consistent inhibition by coelacanth *SERINC*2 (Fig. 3F). Together, the experiments also suggested that the effect of coelacanth *SERINC*2 on nef-defective HIV-1 infectivity inhibition is not cell type specific.

### Gradual loss of antiretroviral activity in *SERINC2*.

**FIG 4:**
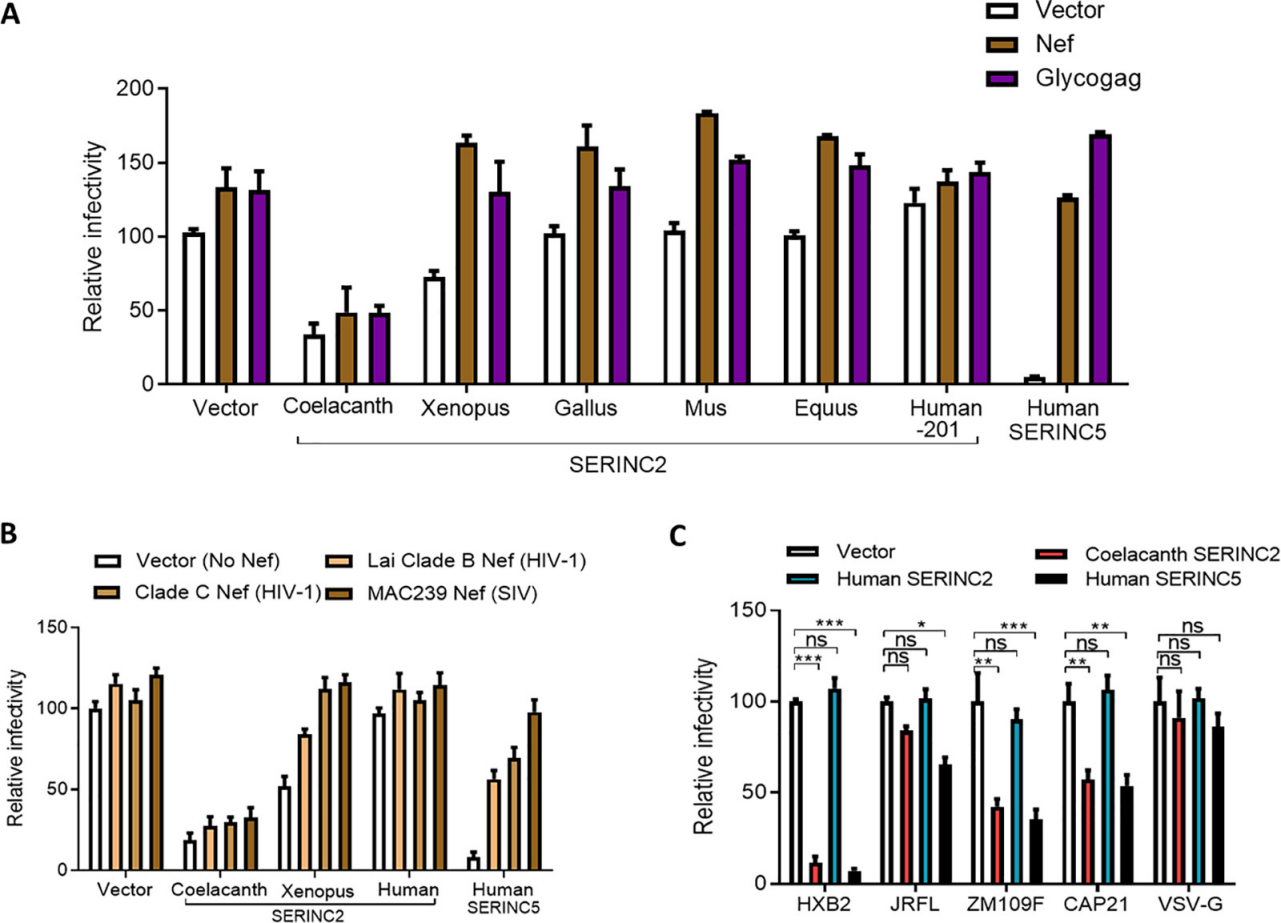
Ability of retroviral factors to antagonize *SERINC*s. (A) A single-cycle infectivity assay was performed using TZM-GFP target cells for HIV-1 particles that were produced from HEK293T cells either by transfecting Nef, glycoGag, or empty vector in in combination with 100 ng of SERINC2 orthologs (coelacanth, *Xenopus*, *Gallus*, *Mus*, *Equus*, and human) and human SERINC5 encoded by PBJ6 or the empty vector backbone along with pHXB2 envelope and NL4-3 Env(−) Nef(−). The infectivity was normalized to RT units using an SGPERT assay. Human *SERINC*5 served as a control for Nef and glycoGag counteraction of the exerted restriction. The values obtained from the empty vector control was set to 100 for comparison with SERINC expressors. Values are means (*n* = 4) and SD. (B) A single-cycle infectivity assay was performed using TZM-GFP target cells for HIV-1 particles that were produced from HEK293T cells by transfecting the indicated Nef alleles or empty vector in in combination with 100 ng of SERINC2 orthologs (coelacanth, *Xenopus*, and human) and human SERINC5 encoded by PBJ6 or the empty vector backbone along with pHXB2 envelope and NL4-3 Env(−) Nef(−). The infectivity was normalized to RT units using an SGPERT assay. The values obtained from the empty vector control were set to 100 for comparison with SERINC expressors. Values are means (*n* = 4) and SD. (C) A single-cycle infectivity assay was performed using TZM-GFP target cells for nef-defective HIV-1 particles that were produced from HEK293T cells by transfecting 100 ng of coelacanth SERINC2, human SERINC2, and human SERINC5 encoded by PBJ6 or the empty vector backbone along with NL4-3 Env(−) Nef(−) with HIV-1 clade B (HXB2 and JR-FL), clade C (ZM109F and CAP210) and vesicular stomatitis virus envelope glycoprotein (VSV-G). The infectivity was normalized to RT units using an SGPERT assay. Human *SERINC*5 served as control for Nef and glycoGag counteraction of the exerted restriction. The values obtained from the empty vector control were set to 100 for comparison with SERINC expressors. Values are means (*n* = 4) and SD. An unpaired *t* test was used. *, *P* < 0.05; **, *P* < 0.01; ***, *P* < 0.001; ns, not significant.

Upon further assessment of the anti-HIV-1 activity of post-WGD *SERINC*2 orthologs, we found that while coelacanth *SERINC*2 reduced the infectivity by ∼7-fold, *Xenopus SERINC*2 exhibited a modest inhibition (∼2-fold). The activity is completely lost in the chicken *SERINC*2 ortholog and onwards (Fig. 3G). However, this lack of activity is persistent in mouse, horse, and human *SERINC*2 at comparable expression levels (Fig. 3G, bottom). Loss of human *SERINC*2 antiviral activity could have been associated with changes in pathogen repertoire or neofunctionalization. To experimentally test if this was in response to a change in the pathogen repertoire, we asked if the counteraction of human *SERINC*5 by known potent retroviral factors, HIV-1 Nef and murine leukemia virus (MLV) glycoGag, was analogous to that of coelacanth *SERINC*2. Under conditions in which Nef and glycoGag efficiently counteracted the restriction exerted by the human *SERINC*5 and the partial restriction of *Xenopus SERINC*2, counteraction of coelacanth *SERINC*2 restriction by these virulent factors was not apparent (Fig. 4A). We used a potent *SERINC*5 antagonist that we reported earlier (simian immunodeficiency virus SIVmac239-encoded Nef) and wondered if this was an allele-specific effect (21). Therefore, we next investigated whether the representative *nef* alleles from human and nonhuman primate lentiviruses showed a similar phenotype and found that nef alleles did not rescue the infectivity in this case as it did for human *SERINC*5 (Fig. 4B). It was also earlier reported by us and others that the ability of *SERINC*5 to restrict HIV-1 inhibition varies with the envelope glycoproteins used for pseudotyping (20, 21). We checked if coelacanth *SERINC*2-mediated inhibition is dependent on the envelope glycoprotein usage. Coelacanth *SERINC*2 action indeed mirrored that of human *SERINC*5 in terms of the envelope sensitivity (Fig. 4C), further highlighting a functional similarity. However, Nef and glycoGag failed to rescue this analogous activity of coelacanth *SERINC*2, indicating a unique feature that perhaps made it invisible to these retroviral factors.

### Human foamy virus envelope counteracts coelacanth *SERINC2*.

**FIG 5:**
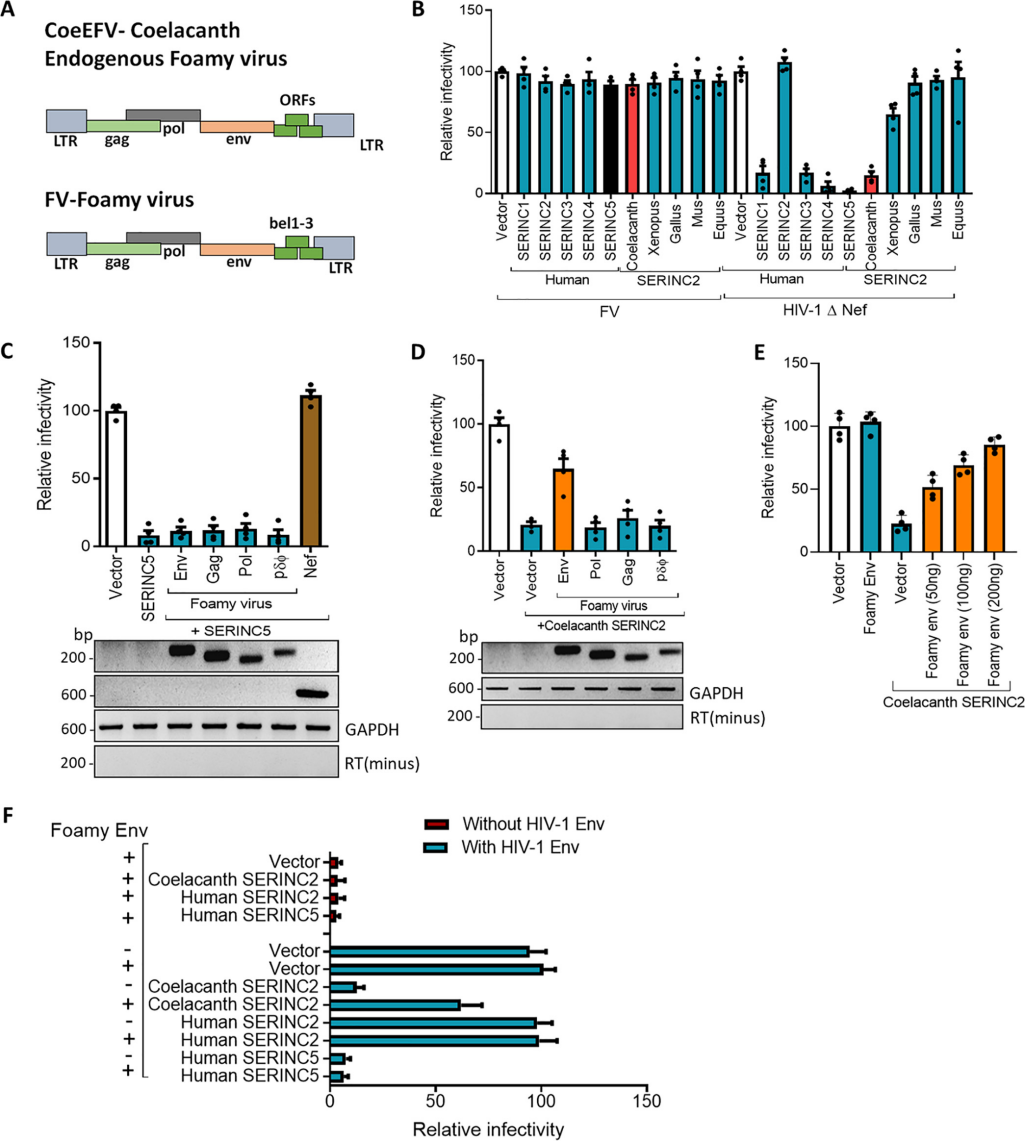
Effect of coelacanth *SERINC*2 on retrovirus infectivity and its antagonism by the envelope glycoprotein. (A) Genome organization of an endogenous foamy virus (CoeEFV) from coelacanth and the prototype foamy virus (FV). (B) A single-cycle infectivity assay was performed using TZM-GFP target cells for FV-derived vectors and nef-defective HIV-1 particles that were produced from HEK293T cells by transfecting 100 ng of SERINC2 orthologs (coelacanth, *Xenopus*, *Gallus*, *Mus*, *Equus*, and human) encoded by pcDNA3.1(−) and human SERINC5 encoded by PBJ6 or the empty vector backbone along with FV-derived vectors for FV and NL4-3 Env(−) Nef(−) and pHXB2 for HIV-1 infectivity. The infectivity was normalized to RT units using an SGPERT assay. Human *SERINC*5 served as a control for Nef and glycoGag counteraction of the exerted restriction. The values obtained from the empty vector control were set to 100 for comparison with SERINC expressors. Values are means (*n* = 4) and SD. (C) (Top) A single-cycle infectivity assay was performed using TZM-GFP target cells for nef-defective HIV-1 particles that were produced from HEK293T cells by cotransfecting FV vectors (env, pol, gag, and transfer vector) and 100 ng of human SERINC5 encoded by PBJ6 or the empty vector backbone along with pHXB2 envelope and NL4-3 Env(−) Nef(−). The infectivity was normalized to RT units using an SGPERT assay. The values obtained from the empty vector control were set to 100 for comparison with SERINC expressors. Values are means (*n* = 4) and SD. (Bottom) RT-PCR for the indicated FV vectors and Nef from transfected cell lysate (HEK293T). GAPDH (glyceraldehyde-3-phosphate dehydrogenase) served as a loading control. The RT(−) control served as a control for amplification from plasmid DNA. (D) A single-cycle infectivity assay was performed using TZM-GFP target cells for nef-defective HIV-1 particles that were produced from HEK293T cells by cotransfecting FV vectors (env, pol, gag, and transfer vector) and 100 ng of coelacanth SERINC2 encoded by pcDNA3.1(−) or the empty vector backbone along with pHXB2 envelope and NL4-3 Env(−) Nef(−). The infectivity was normalized to RT units using an SGPERT assay. The values obtained from the empty vector control were set to 100 for comparison with SERINC expressors. Values are means (*n* = 4) and SD. (Bottom) RT-PCR for the indicated FV vectors and Nef from transfected cell lysate (HEK293T). GAPDH served as a loading control. The RT(−) control served as a control for amplification from plasmid DNA. (E) A single-cycle infectivity assay was performed using TZM-GFP target cells for nef-defective HIV-1 particles that were produced from HEK293T cells by cotransfecting increasing amounts of FV env and 100 ng of coelacanth SERINC2 encoded by pcDNA3.1(−) or the empty vector backbone along with pHXB2 envelope and NL4-3 Env(−) Nef(−). The infectivity was normalized to RT units using SGPERT assay. The values obtained from the empty vector control were set to 100 for comparison with SERINC expressors. Values are means (*n* = 4) and SD. (F) A single-cycle infectivity assay was performed using TZM-GFP target cells for nef-defective HIV-1 particles that were produced from HEK293T cells by transfecting 100 ng of coelacanth SERINC2, human SERINC2 encoded by pcDNA3.1(−), human SERINC5 encoded by PBJ6, or the empty vector backbone along with pHXB2 envelope and NL4-3 Env(−) Nef(−) in the presence and absence FV env. The infectivity was normalized to RT units using SGPERT assay. The values obtained from the empty vector control was set to 100 for comparison with SERINC expressors. Values are means (*n* = 4) and SD.

Three distinct retroviruses were reported to have independently come up with antagonizing factors to elude the inhibition by *SERINC*5 (20–22, 35). The lack of activity in Nef and glycoGag against coelacanth *SERINC*2 (Fig. 4A and B) prompted us to check whether antiretroviral SERINC2 has evolved to inhibit other retroviruses. We learnt that the coelacanth has an endogenous foamy virus (36), the genome organization of which resembles that of the prototype foamy virus (FV) (Fig. 5A). Recent work has also shown the existence of endogenous foamy virus in reptiles, birds, and various mammals (37, 38). To experimentally test the presence of anti-FV activity in human *SERINC* paralogs as well as the *SERINC*2 orthologs, we first tested the activity of all human SERINCs against a human FV. Surprisingly, we find that FV is insensitive to any of the human *SERINC* paralogs tested as well as the coelacanth *SERINC*2, under conditions in which nef-defective HIV-1 was consistently inhibited (Fig. 5B). We argued that this might have been associated with the intrinsic ability of an FV-encoded factor to counter the inhibition. To delineate this, we coexpressed foamy virus genes individually to check their ability to rescue nef-defective HIV-1, so that the insensitivity of FV and the presence of antagonizing factor could be revealed. Surprisingly, with none of the FV components expressed in *trans*, we found an ability to rescue the inhibition exerted by human *SERINC*5. Under these conditions, however, Nef completely antagonized the *SERINC*5 restriction (Fig. 5C). The inhibition exerted by coelacanth *SERINC*2, interestingly, was antagonized by the FV envelope glycoprotein (Fig. 5D). The phenotype was also consistent when we expressed increasing amounts (50, 100, or 200 ng) of FV envelope (Fig. 5E). Due to the unavailability of commercial antibodies against FV vectors, we attempted to tag the FV envelope glycoprotein with HA and FLAG for visualizing the expression by Western blotting. However, the C-terminal epitope tagging resulted in noninfectious FV particles (data not shown), suggesting interference of the tag with envelope function. We reasoned that the constructs were valid, as they consistently contributed to the production of functional virus particles in the infectivity assays, which was considered proof of expression for foamy components. Regardless, we confirmed the mRNA-level expression using specific primers for the indicated FV genes and found that they were expressed in these experimental settings (Fig. 5C and D, lower panels).

Having found an envelope counteracting the SERINC2 activity, we further investigated whether this rescue was an effect of cross-packaging of the HIV-1 core by the FV envelope. By using a pseudoparticle comprising an HIV-1 core complemented with the FV envelope, we showed that the effect was not due to cross-packaging (Fig. 5F), as the FV envelope incorporation into budding virions would yield infectious particles under these conditions, which was not the case. This rescue by the FV envelope glycoprotein, therefore, appears to be specific to its ability to antagonize coelacanth *SERINC*2, and thereby, it restores most of the HIV-1 infectivity (Fig. 5D).

### FV envelope prevents *SERINC2* incorporation into virions.

**FIG 6:**
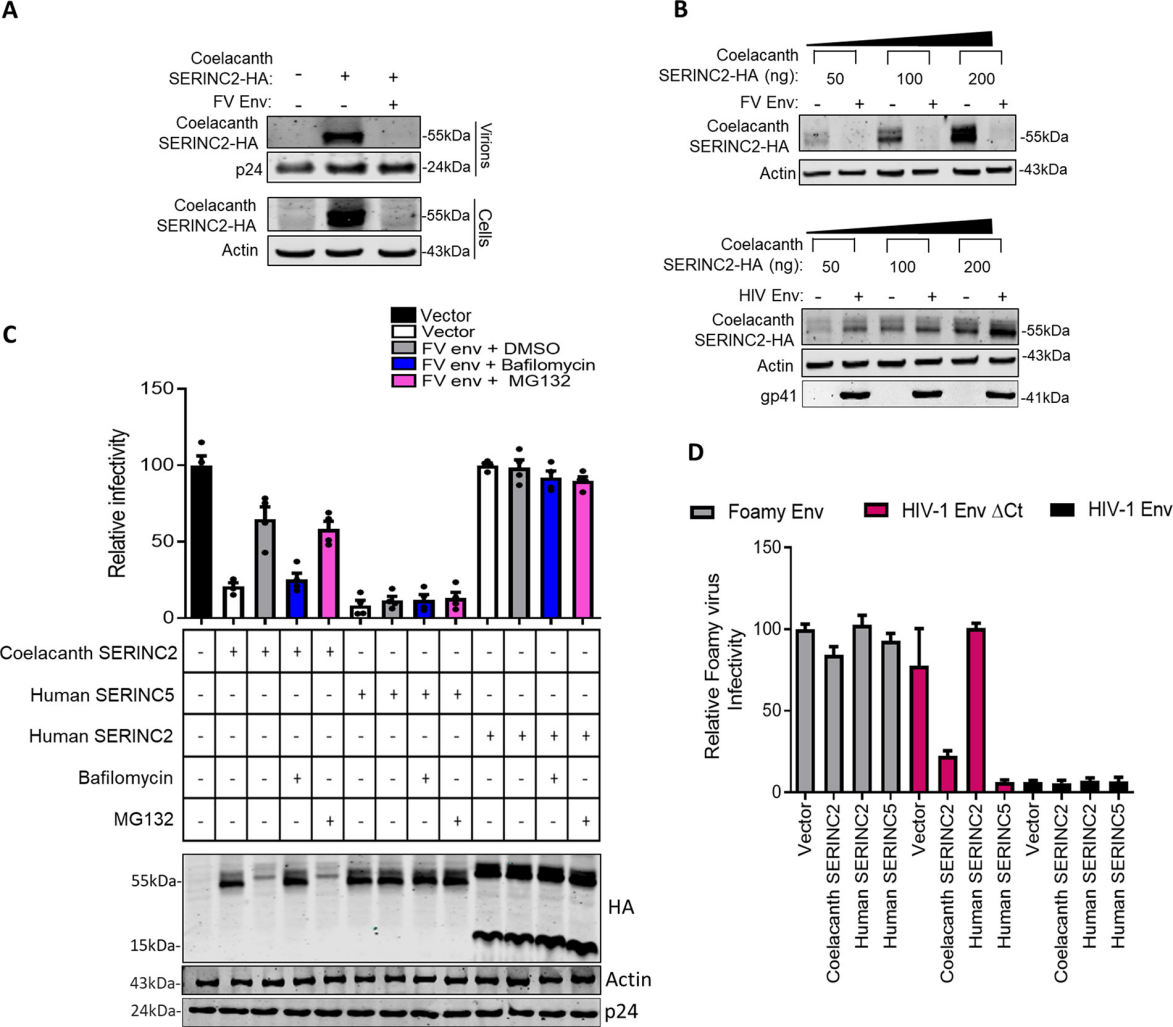
FV envelope targets coelacanth *SERINC2* for degradation. (A) (Top) Western blot of HA-tagged coelacanth *SERINC*2 with the corresponding p24 from virions in the presence and absence of foamy virus envelope. (Bottom) Western blot of HA-tagged coelacanth *SERINC*2 with corresponding β-actin from HEK293T cell lysate. (B) (Top) Western blot of HA-tagged coelacanth *SERINC*2 at 50 ng, 100 ng, and 200 ng in the presence (+) and absence (−) of FV envelope (200 ng) and corresponding β-actin from HEK293T cell lysate from a 12-well plate. (Bottom) Western blot of HA-tagged coelacanth *SERINC*2 at 50 ng, 100 ng, and 200 ng in the presence (+) and absence (−) of HIV-1 envelope and corresponding β-actin and HIV-1 gp41 from HEK293T cell lysate from a 12-well plate. (C) (Top) A single-cycle infectivity assay was performed using TZM-GFP target cells for nef-defective HIV-1 particles that were produced from HEK293T cells by transfecting 100 ng of coelacanth SERINC2, human SERINC2 encoded by pcDNA3.1(−), human SERINC5 encoded by PBJ6, or the empty vector backbone along with pHXB2 envelope and NL4-3 Env (-) Nef(−) in the presence or absence of FV env. In the presence of FV env, the cells were treated with either DMSO control, bafilomycin A1 (100 nM), or MG132 (20 μM) for 15 h. The infectivity was normalized to RT units using an SGPERT assay. The values obtained from the empty vector control were set to 100 for comparison with SERINC expressors. Values are means (*n* = 4) and SD. (Bottom) Western blot showing expression of C-terminally HA-tagged *SERINC* expressors for indicated conditions and the corresponding β-actin and HIV-1 p24 from cell lysate (HEK293T). (D) A single-cycle infectivity assay was performed using TZM-GFP target cells for FV particles produced from HEK293T cells. The foamy core was pseudotyped with either FV envelope, HIV-1 envelope, or HIV-1 envelope lacking a C-terminal tail (ΔCt), and the indicated coelacanth SERINC2, human SERINC2 encoded by pcDNA3.1(−), human SERINC5 encoded by PBJ6, or the empty vector backbone was coexpressed to check the ability of individual *SERINC* to restrict foamy virus. Values are means (*n* = 4) and SD.

We previously established that virulence factors like Nef, S2, and glycoGag promoted the exclusion of *SERINC*5 from virions to increase the particle infectivity (20, 21). We investigated whether the FV envelope glycoprotein acts similarly. Nef-defective particles were produced from HEK293T cells in the presence of coelacanth *SERINC*2 and FV envelope glycoprotein. Western blotting revealed that although coelacanth *SERINC*2 was readily detectable in the nef-defective virions, the expression of FV envelope diminished the signal to a level similar to the background (Fig. 6A). This experiment indicated that, like Nef, S2, and glycoGag, the FV envelope enhances the particle infectivity by preventing the virion incorporation of the coelacanth *SERINC*2.

Strikingly, we observed that when FV envelope was coexpressed, the steady-state level of coelacanth *SERINC*2 in the producer cell lysates was affected (Fig. 6A, Western blots from producer cells). This indicates that FV envelope affects the stability of coelacanth *SERINC*2 by promoting its degradation. To examine this further, we tested the effect of FV envelope in HEK293T cells by expressing increasing amounts of coelacanth *SERINC*2 (50, 100, and 200 ng) against a constant amount of FV envelope (100 ng) glycoprotein-encoding plasmid. Western blotting revealed that coexpression of FV envelope glycoprotein resulted in a decreased amount of coelacanth *SERINC*2 in cell lysates regardless of the amount of plasmid transfected (Fig. 6B). In contrast to the effect of FV envelope observed on coelacanth *SERINC*2, the steady-state level of the protein remained unperturbed when it was coexpressed with an HIV-1 envelope glycoprotein under similar experimental conditions (Fig. 6B, bottom).

Interestingly, we found that FV envelope decreases steady-state levels of coelacanth *SERINC*2 in a bafilomycin-sensitive manner, implying that coelacanth *SERINC*2 is targeted to lysosomes for destruction. Accordingly, the ability of FV env to rescue the infectivity is impaired upon challenge with bafilomycin during the virus production but not by the proteasomal inhibitor MG132 (Fig. 6C). Notably, we did not see any apparent change in the expression levels of human *SERINC5* or *SERINC2* upon FV envelope expression (Fig. 6C, bottom). Accordingly, the infectivity inhibition exerted by *SERINC5* is not counteracted by FV envelope glycoprotein, which is consistent with the data in Fig. 5C. Further, bafilomycin A1 (lysosomal acidification inhibitor) and MG132 (proteasomal inhibitor) did not affect the outcome of infectivity in the case of human *SERINC2*. Taken together, these experiments suggested that the effect of FV envelope on the steady-state levels of coelacanth *SERINC*2 is specific.

### Sensitivity of FV to coelacanth *SERINC2*.

Further, we questioned whether the foamy virus was at all sensitive to the effects of coelacanth *SERINC*2. To this end, we pseudotyped FV core with an envelope glycoprotein of HIV-1 lacking a C-terminal tail, because the native full-length HIV-1 envelope was not capable of packaging the core to produce infectious virions (Fig. 6D). Using a HIV-1 envelope with a C-terminal deletion, we showed that the FV core is sensitive to coelacanth *SERINC*2 restriction (Fig. 6D). Altogether, this indicates that the FV core is susceptible to the action of coelacanth *SERINC*2, and the virus possesses a mechanism to antagonize the inhibitory effect.

## DISCUSSION

The use of comparative evolutionary genetic approaches has continued to enrich our understanding of restriction factor biology for more than a decade (7, 39–42). Reciprocal loss of duplicated genes in different species has been shown to contribute toward species-specific differences in susceptibility to pathogens (43–45). We found that, similar to reciprocal gene loss, *SERINC*2 and *SERINC*5 show reciprocal functional adaptation against divergent retroviruses. While the spectrum of activity among human paralogs we tested here is in agreement with previous findings (21, 22, 24, 25), the ability of *SERINC* orthologs to restrict HIV-1 is also remarkable. Since exogenous retroviruses have not yet been reported from pre-WGD species that we considered, the range of targets that a pre-WGD *SERINC* ortholog (TMS-1) will have remains to be ascertained. While genes such as CCR4 and DHH1 that physically interact with TMS-1 have been implicated in yeast retrotransposon activity (46, 47), the mechanism that TMS-1 would manifest on retroelements, if any, remains to be investigated. TMS-1 in flies may still be required to regulate the mobility of gypsy retroelements, as the gypsy envelope has been used to pseudotype Moloney murine leukemia virus-based vectors to efficiently infect fly cells (48, 49).

**FIG 7:**
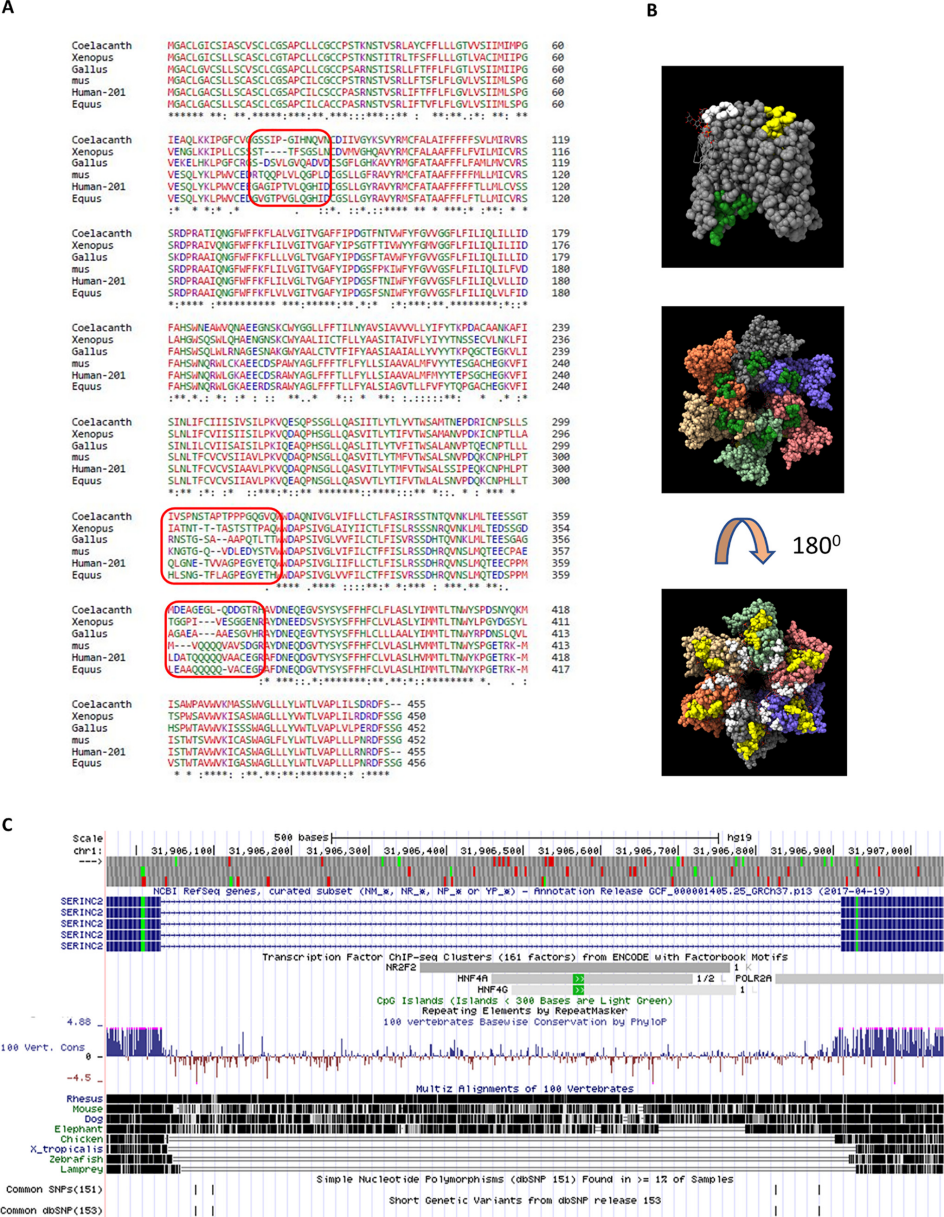
Sequence divergence among SERINC2 orthologs and structural analysis. (A) Multiple-sequence alignment (MSA) of *SERINC*2 orthologs using ClustalW; highlighted regions are the sites of sequence divergence. (B) Cryo-EM structure of TMS-1 from D. melanogaster reported in reference 56, visualized using ChimeraX software. The regions highlighted in the MSA in panel A are in white (first region), yellow (second region), and green (third region). (Top) Monomer; (middle) hexamer front view; (bottom) hexamer rear view. (C) Evidence for the presence of an HNF4a binding site in the intron of the human SERINC2 gene.

Moreover, the analogous mechanism that Rous sarcoma virus uses to produce Pr180gag-pol (as found in yeast Ty-1 transposons [50]) makes the existence of retroelement interactions for other host factors like TMS-1 more probable. Post-WGD, *SERINC* has five copies, and this may have been associated with tackling an increasing diversity of pathogens during speciation while simultaneously retaining core functionality. As demonstrated here with cross-packaging studies, we show that a *SERINC*2 ortholog—which was thought to be inactive—may have constituted a critical barrier to foamy viruses early on, plausibly leading to their divergence (36, 51). Loss of activity in other *SERINC*2 orthologs may have been associated with neofunctionalization, as suggested by localized sequence divergence (Fig. 7A and B; also, see Videos S1 and S2 in the supplemental material), the presence of HNF4alpha binding enhancer (Fig. 7C), and changes in tissue-specific expression patterns (see Data Set S1) exemplified by expression of *SERINC*2 in the livers of primates. For the first time, we observed a naturally occurring antiretroviral *SERINC2* from coelacanth. Future studies exploring the sequence level differences shown in Fig. 7B (also see Videos S1 and S2) among functionally distinct *SERINC2* orthologs may help in dissecting the antiretroviral function from its putative tissue-specific function. Interestingly, coelacanth *SERINC*2 inhibited HIV-1 and remained invisible to the most potent retroviral virulent factors, Nef (SIVmac239) and the glycoGag from MLV. Further studies can provide more insights into the lack of activity in these retroviral factors and require relevant models to study physiological relevance.

Foamy viruses codiverged with their hosts (36, 38, 52), and this interrelation is visible in their ability to introduce variations in envelope glycoproteins, among other documented genomic alterations. The FV envelope counteracting *SERINC*2 is the first evidence of a canonical gene product being employed to evade *SERINC*2 restriction. The FV envelope’s ability to engage the endolysosomal machinery of the host to selectively target coelacanth *SERINC*2, but not human *SERINC2* and *SERINC5*, for degradation in a heterologous host is impressive, as was the case with other retroviral factors (53, 54). The counteraction phenotype associated with the FV envelope demonstrated here is also in agreement with the ability of *SERINC*5 to restrict the virus in an envelope-dependent manner (20, 21, 25, 31, 55, 56), indicating the envelope as a determinant for *SERINC* sensitivity to particle restriction. Therefore, the finding of FV envelope-dependent activity against coelacanth SERINC2 is in agreement with previous reports (55, 56). We acknowledge that for the study of foamy viruses and their interaction with coelacanth *SERINC*2, the absence of a native experimental model was a limitation. The availability of such experimental models would have strengthened the findings further to understand the role of *SERINC*s in shaping the evolution of retroviruses other than HIV-1. Further, no exogenous retrovirus is known to infect coelacanths (36). A better understanding of SERINC activity against foamy viruses therefore awaits discovery of new exogenous foamy viruses.

Similarly, the role of *SERINC*5 from coelacanths in inhibiting retrovirus remains to be established due to the unavailability of reliable genomic sequence information to reconstruct a functional protein-coding gene. An early challenge by *SERINC*2 may have led to subsequent divergence of the FV envelope toward insensitivity to *SERINC2* restriction. Different *SERINC* paralogs, therefore, might have specialized in restraining specific retroviruses, leading to the coevolution in response to specific paralogs (32, 57).

We foresee that weaker signatures, exemplified by *SERINC*s despite the constant challenge from viruses, could be due to the native functions of such transmembrane proteins, where adaptation through diversification in response to the pathogen would result in the loss of a core function (58). The poor signatures could also be because the antiretroviral activity is spread out over multiple host genes; for instance, a recent report shows that a Nef-sensitive TIM1 activity is potentiated by *SERINC*5 (59). The host, therefore, can afford such redundancy without having to diversify much. Another example is the TLRs, where the extracellular domain shows signatures of recurrent positive selection in contrast to the conserved membrane-spanning region (15). Intriguingly, virus-specific TLRs are under stronger purifying selection than nonviral TLRs (60), potentially due to the larger number of pathogen-associated molecular patterns (PAMPs) associated with nonviral pathogens. This constraint, however, may be more pronounced in *SERINC*s, as they are multipass transmembrane proteins known to inhibit only retroviruses. While the core function of *SERINC*s in eukaryotes awaits independent observations (61), our study indicates more *SERINC*-like restriction factors that display poor signatures of arms race but are functionally active against viruses.

In conclusion, evolution-guided analysis for tracking the origin of antiviral activity in the *SERINC* genes and the dynamics following whole-genome duplication have identified the presence of antiretroviral activity in the only *SERINC* thought to be deficient. The antiretroviral activity among *SERINC*s may have shaped the evolution of distant retroviruses, and the presence of an evasion strategy in a spumavirus representative to target coelacanth *SERINC*2 implies a fundamental role of these host factors in shaping retrovirus evolution.

## MATERIALS AND METHODS

### Plasmids and reagents.

The plasmids and reagents, including antibodies used in the current study, are presented in Tables SI and SII, respectively.

### Viruses and infectivity assay.

Viruses were produced from HEK293T (ECACC) cells (21) plated in 10-cm^2^ dishes by calcium phosphate transfection and were limited to single-cycle replication. Seven micrograms of NL4-3 (env defective and Nef defective), 1 μg env-expressing plasmid, and 100 ng of plasmids expressing *SERINC* genes (Table SI) or the equivalent corresponding empty vector and PBJ6 *SERINC*5 HA were used for virus production. Foamy viruses were produced by transient transfection using pCIES (0.736 μg), pCIPS (1.5 μg), pCIGS (11.84 μg), pΔΦ (11.84 μg) (from reference 62; plasmid details are provided in Table SI) and pcDNA (100 ng) vectors expressing *SERINC* genes or an equivalent amount of control vector in HEK293T cells. The viral particles were collected as cell culture supernatant 48 h posttransfection, centrifuged at 300 × *g* for 5 min, and quantified using an SG-PERT reverse transcription assay (63, 64). Following this, viruses were diluted for infection in TZM-GFP reporter cells (21) that were seeded in a 96-well plate, 24 h prior to infection. HIV-1 infectivity was quantified by scoring the green nuclei using a SpectraMax MiniMax 300 imaging cytometer (Molecular Devices, USA). Foamy virus infectivity was examined by determining the number of green fluorescent protein (GFP)-positive cells expressed from the transducing vector carrying a GFP expression cassette (pΔΦ), indicating the fraction of the cell population transduced. The acquired values were normalized to the reverse transcriptase units obtained from the SG-PERT assay as previously described (20). Results are expressed as percentages of the value for a vector control, normalized to 100.

### Immunofluorescence.

For electroporation of JTAg*^SERINC^*^3/5KO^ (20), cells in exponential growth phase were harvested (10^7^ cells/sample) at 300 × *g* for 10 min. Prior to addition of Opti-MEM, the cells were washed with 1× phosphate-buffered saline (PBS [1×], pH 7.0) to remove residual serum and cell debris. Each sample was resuspended in 200 μl warm Opti-MEM. Five micrograms of constructs expressing HA-tagged *SERINC*2 orthologs or equivalent control vector was then mixed into the suspended cells. The cells and DNA mixture were added to a 2-mm-gap electroporation cuvette (Bio-Rad, USA). The cells were pulsed at 140 V and 1,000 μF with exponential decay on a Bio-Rad GenePulserXcell module. Warm RPMI (600 μl) with 20% fetal bovine serum (FBS) was immediately added to the electroporated cells, which were then transferred to a 6-well plate containing RPMI with 10% FBS. Forty-eight hours posttransfection, cells were spun down at 300 × *g* for 5 min, resuspended in 100 μl RPMI, laid on poly-l-lysine-coated glass coverslips (Genetix), and fixed with 4% paraformaldehyde (PFA). After fixation, the cells were washed twice with 1× PBS. The cells were then permeabilized using BD Perm/Wash followed by detection of HA tag with purified anti-HA.11 epitope tag antibody (1:200) and Alexa Fluor 488-tagged secondary antibody (1:500). The coverslips were transferred to a glass slide and mounted using ProLong glass antifade reagent. Images were acquired after 12 h with a Zeiss confocal microscope (LSM740).

### Incorporation of SERINCs into virions.

Viruses were produced by transfecting HEK293T cells using calcium phosphate transfection reagent in a 10-cm^2^ plate, as mentioned above. The medium was replaced with Dulbecco’s modified Eagle medium (DMEM) containing 2% FBS after 12 to 15 h of transfection. After 48 h, the virus-containing supernatant was collected and centrifuged at 500 × *g* for 10 min to exclude any cell debris. Following this, the viruses were filtered using a 0.22-μm syringe filter (Cole Parmer). The suspension was overlaid on a 25% sucrose (in 1× PBS) cushion and concentrated at 100,000 × *g* for 2 h at 4°C using a Beckman-Coulter ultracentrifuge. After the spin, the supernatant was aspirated, and the pellet was suspended in Laemmli buffer containing 50 mM Tris(2-carboxyethyl) phosphine hydrochloride (TCEP).

### Western blotting.

After the collection of viruses from producer cells (HEK293T), the cells were harvested in ice-cold PBS. Cells were washed twice at 500 × *g* for 5 min. The PBS was aspirated until the pellet was completely dry. The pellets were either processed for lysis or stored in a −80°C freezer until further application. The cell pellets were lysed in DDM lysis buffer (100 mM NaCl, 10 mM HEPES [pH 7.5], 50 mM TCEP, 1% *n*-dodecyl-β-d-maltoside [DDM]) and EDTA-free protease inhibitor cocktail and rocked on ice for 30 min. Following this, the lysates were clarified by centrifugation at 10,000 × *g* for 15 min, and the supernatant was collected and mixed with 4× Laemmli buffer with 50 mM TCEP.

SDS-Tricine-PAGE was used for resolving cell pellets and virions for analysis by Western blotting. The proteins from the gel were electrotransferred onto a low-fluorescence polyvinylidene difluoride (PVDF) membrane using a semidry transfer unit (TE77; Hoefer, USA) for 75 min with a 125-A constant current with the voltage set at a maximum of 20 V. The membrane containing proteins was then blocked with Odyssey blocking buffer. After 20 min of blocking, anti-HA.11 epitope tag antibody (mouse monoclonal) and anti-actin (rabbit monoclonal) (diluted 1:4,000 in blocking buffer) were used to probe the membrane to detect the HA-tagged proteins. After incubation with the primary antibody for 1 h, the membrane was washed three times with 1× Tris-buffered saline–Tween (TBS-T) for 5 min. Goat anti-mouse immunoglobulin (680RD) and goat anti-rabbit immunoglobulin (800CW; Li-Cor) antibodies were used to detect infrared dye signal from the membrane. The Western blot signal was acquired and analyzed on the Odyssey imager system (Li-Cor Biosciences) and is presented in greyscale format.

### RT-PCR.

For reverse transcription-PCR (RT-PCR), HEK293T cells were cotransfected with FV vectors (env, pol, gag, and transfer vector) and 100 ng of human SERINC5 or coelacanth SERINC2 along with pHXB2 envelope and NL4-3 Env(−) Nef(−) for production of nef-defective HIV-1. The producer cells were then collected for RNA isolation using TRIzol followed by DNase treatment to remove plasmid DNA contamination. The RNA was then concentrated using an RNA cleanup and concentrator kit (Thermo Scientific). From this, first-strand cDNA synthesis was performed using reverse transcriptase (Thermo Scientific). The cDNA was then diluted and used as a template for the amplification of FV vectors using the following primers: FV env forward, 5′-CCGGAACCCATAGTGGTGAA-3′; FV env reverse, 5′-CTTAGGCCACGGTTTGGGT-3′; FV pol forward, 5′-CTGGGATTCAGGGGCAACAA-3′; FV pol reverse, 5′-CCAAGGAACATCTGTTGGCG-3′; FV gag forward, 5′-CTTCCTGCTCCTGTACCGTC -3′; FV gag reverse, 5′-AGGAGCATTTCGTCCTAGCC-3′; FV transfer vector (pdeltaphi) forward, 5′- GACGGTATCGATGGTACCGG -3′; FV transfer vector (pdeltaphi) reverse, 5′- TTAAGAACCTTGTGTCTCTC -3′; Nef (SIVmac239) forward, 5′- ATGGGTGGAGCTATTTCCAT -3′; Nef (SIVmac239) reverse, 5′- GCGAGTTTCCTTCTTGTCAG -3′; GAPDH forward 5′- TGGAGAAGGCTGGGGCTCATTTGCA -3′; GAPDH reverse 5′- CATACCAGGAAATGAGCTTGACAA-3′.

The PCR conditions were 95°C for 2 min (initial denaturation), followed by 95°C for 30 s (final denaturation), 55°C for 30 s (annealing), and 74°C for 1 min (extension) for 30 cycles, and 74°C for 10 min (final extension).

### Genome correction and quality check.

It has been shown that the genomic sequences for many high-quality primate genomes provided in databases such as NCBI have artifactual single-base-pair-level errors (65). For example, the genome assembly sequence available in the database might have the incorrect base “A” at a particular site. However, the actual base present in the DNA of the organism might be the base “T.” Such artifacts in the genomic sequence get introduced during the sequencing error correction step of the genome assembly process. Despite the various biases (66, 67) that result from such poor-quality sequences, the error-prone sequences continue to be a source of incorrect and misleading results (68).

To rule out the possibility of artifacts in the evolutionary analysis that arise from errors in the sequences of genome assemblies (true even for high-quality reference genomes found on Ensembl [68]), we systematically assessed the quality of each of the primate genomes used in this study. Genome assemblies of 15 primate species were downloaded from Ensembl release 98 through the FTP (file transfer protocol) site. Whole-genome-sequencing data sets corresponding to each of these species were obtained from the Short Read Archive (SRA) with the criterion of ≥30× coverage. Details of the genome assemblies used and the corresponding raw read data from each species are provided in Table SIII. The raw read data were mapped to the corresponding genomes using the bwa mem read mapper (69) with default settings. The alignment files obtained from the mapping step were used to generate the genotype likelihood estimates using the program angsd (70). The genotype likelihood estimates were provided to the program referee (71) to assign quality scores and perform genome correction. Overall, we found that in all the primate genomes considered, less than 1% of the bases were corrected by the program referee. The sequencing data used for performing genome correction are not from the same individual that was used for genome assembly. Hence, it is possible that many of the corrected positions are merely nucleotide polymorphisms. These quality control steps ensured that none of the arms race signatures detected in the genes considered in our study is the result of incorrect genomic sequences.

### Manual curation and multiple-sequence alignment.

The manually curated open reading frame multi-fasta files consisting of 70 genes from ∼15 primate species were collected from Ensembl. The 15 primate species from the infraorder Simiiformes used to compare the arms race signatures consists of olive baboon (Papio anubis), common marmoset (Callithrix jacchus), African green monkey (Chlorocebus sabaeus), gorilla (Gorilla gorilla *gorilla*), human (Homo sapiens), rhesus macaque (Macaca mulatta), crab-eating macaque (Macaca fascicularis), bonobo (Pan paniscus), chimpanzee (Pan troglodytes), golden snub-nosed monkey (Rhinopithecus roxellana), squirrel monkey (Saimiri boliviensis *boliviensis*), drill (Mandrillus leucophaeus), sooty mangabey (Cercocebus atys), Nancy Ma’s night monkey (Aotus nancymaae), and Panamanian white-faced capuchin (Cebus capucinus *imitator*). The species name and Ensembl transcript ID for each of the genes used to look for arms race signatures are provided in Table SIV. We extended our previous multiple-sequence alignment strategies (72) by including additional multiple-sequence alignment programs to generate eight independent alignments for each gene. Several multiple-sequence alignment tools were used to ensure that the inferred patterns of sequence evolution were not restricted to the alignment strategy used. The choice of the actual multiple-sequence alignment tools used was based on the performance-based classification of algorithms (73).

### Use of FUBAR to find evolutionary fingerprints.

Traditional approaches that endeavor to find arms race signatures in genes look for the recurrent occurrence of positive selection in the same gene in different evolutionary lineages. However, when arms race signatures need to be estimated and compared for large numbers of species, such lineage-specific tests are time-consuming and result in reduced statistical power. A more recent approach has been to use the full joint distribution of synonymous (α) and nonsynonymous (β) rates as an evolutionary fingerprint of a gene (19). The program FUBAR (74) is available as part of the HyPhy package (75). FUBAR quantifies evolutionary fingerprints of a gene by dividing the synonymous (α) and nonsynonymous (β) rates into a finely discretized grid. Each point in this grid represents a pair of α and β values, with the size of the circle representing the value θ̂. The value θ̂ is the posterior mean, which provides a measure of how many of the sites in the multiple-sequence alignment contribute to that particular combination of α and β values. The vector of 400 θ̂ values that correspond to each of the points on the 20-by-20 grid of α and β values serves as the evolutionary fingerprint of a gene. Genes with canonical arms race signatures have a greater weight for grids with higher values of β than genes that lack the arms race signature (19). The use of such signatures to quantify arms race signatures is convenient for comparing signatures of several genes. Previous comparisons of these signatures in primates have shown that the well-known arms race genes BST-2, APOBEC3F, APOBEC3G, and TRIM5-alpha have similar signatures of arms race that are missing in SERINC3 and SERINC5 (19). However, the genes SERINC1, SERINC2, and SERINC4 were not used in the previous study. To better understand the similarities and differences in the evolutionary signatures among the SERINC genes with respect to other known restriction factors, we used the hierarchical clustering of the arms race signatures of all these genes (Fig. 1C). We calculated the distance measure defined by Murrell et al. (19) to perform the hierarchical clustering of the selection signatures obtained from FUBAR.

### Rationale for using coelacanth SERINC2.

More than 95% of all extant fish species belong to the infraclass Teleostei (teleost fish), and abundant fish species that are commercially important or serve as model organisms (such as the zebrafish) belong to this group (76). These teleost fish belong to the class Actinopterygii (ray-finned fishes); they actually contain six copies of the SERINC genes and are known to have had a third round of whole-genome duplication (3R-WGD) (77). Phylogenetic studies have consistently found that the coelacanths and lungfish belong to the clade Sarcopterygii (lobe-finned fish) and share a more recent common ancestor with tetrapods than Actinopterygii (78). Importantly, the coelacanths share the two rounds of whole-genome duplication (2R-WGD) found in other tetrapods and lack the third round (3R-WGD) of whole-genome duplication found in teleost fish (77). Among the currently available high-quality genome assemblies, the coelacanth is the only organism from the clade Actinopterygii, and it has five copies of the SERINC genes. Therefore, we selected the coelacanth for our studies on SERINC2.

### Visualization of TMS-1 structure.

Most recently, the structure of pre-WGD SERINC (TMS-1) was resolved by cryo-electron microscopy (cryo-EM) and was found to consist of six monomers forming a hexamer structure (56). We downloaded this structure from PDB (Protein Data Bank; accession number 6SP2) and visualized the broad regions (Fig. 7B) corresponding to these three clusters of sequence divergence (Fig. 7A) using ChimeraX software (79). The first region, corresponding to the D. melanogaster TMS-1 protein sequence MPFCINSTSSYSSGALSAVSGGSLQ, is highlighted in white; the second region, with the sequence TWSAVANNPEKECNPGMFGMMEGFGNATTTAAPPTHTTRVTFDTTNIIG, is highlighted in yellow; and the third region, CISSAVEVSKISHDNSEKRDTEAGTDGSGKPSTDTETEGVTYSWS, is highlighted in green.

### GenBank accession numbers for genes used in experimental studies.

The GenBank numbers for genes used here are as follows: Saccharomyces cerevisiae TMS-1, NM_001180413; Drosophila melanogaster TMS-1, NM_140636.3; Xenopus tropicalis SERINC5, XM_002940195, and SERINC2, NM_001016562.2; Gallus gallus SERINC5, XM_424762.6, and SERINC2, NM_001030890.1; Mus musculus SERINC5, NM_172588.2, and SERINC2, NM_172702.3; Equus caballus SERINC5, XM_001503874.4, and SERINC2, XM_023634619.1; Homo sapiens SERINC1, NM_020755.4, SERINC2, NM_001199038.2, SERINC2 short isoform, NM_178865.5, SERINC3, NM_006811.4, SERINC4, NM_001258031.1, and SERINC5, NM_001174072.3; and coelacanth (Latimeria chalumnae) SERINC2, XM_005993129.2,

### Statistical analysis.

The significance of results was statistically analyzed using Student’s unpaired *t* test in GraphPad Prism 9. The relative differences were considered statistically significant when *P* was <0.05.
