# Supplementary material for: Coelacanth SERINC2 Inhibits HIV-1 Infectivity and Is Counteracted by Envelope Glycoprotein from Foamy Virus
Source: J Virol. 2021 Jun 10;95(13):e00229-21. doi: 10.1128/JVI.00229-21 (PMC8316019; doi:10.1128/JVI.00229-21)
Supplement: Supplemental file 1 — Tables SI to SIV; Supplemental Legends; Data Set S1. Download JVI.00229-21-s0001.pdf, PDF file, 721 KB [file jvi.00229-21-s0001.pdf]

Supplemental material

SI Table I: List of Plasmids used in this study

S.No	Plasmid name	Purpose	Notes	Source
1.	NL4-3 E(fs) N (fs)	A plasmid for expression HIV-1 Laboratory Strain lacking Nef and Env ORFs	Defective in Env and Nef	Rosa et al 2015, NIH AIDS Reagent Program
2.	pHXB2 SRAlpha	A plasmid for expression of HIV-1 HXB2 envelope of clade B	SRα promoter driven HIV envelope	Rosa et al 2015, NIH AIDS Reagent Program
3.	pcDNA 3.1 (-) SERINC1 CO HA	Expression of Codon-optimized Human SERINC1	C-terminal HA Tag	This study
4.	pcDNA 3.1 (-) SERINC2 HA	Expression of Human SERINC2	C-Terminal HA Tag	[1]Sood et al 2017
5.	pcDNA 3.1(-) SERINC4 CO HA	Expression of Codon-optimized Human SERINC4	C-Terminal HA Tag	This study
6.	pcDNA 3.1 (-) Coelacanth SERINC2 HA	Expression of Codon-optimized Coelacanth SERINC2	C-Terminal HA Tag	This study
7.	pcDNA 3.1(-) Human SERINC2 -201 HA	Expression of shorter isoform of Human SERINC2	Human SERINC2 (short)	This study
8.	pcDNA 3.1(-) Xenopus SERINC2 HA	Expression of Codon-optimized Xenopus SERINC2	C-Terminal HA Tag	This study
9.	pcDNA 3.1(-) Gallus SERINC2 HA	Expression of Codon-optimized Gallus SERINC2	C-Terminal HA Tag	This study
10.	pcDNA 3.1(-) Mus SERINC2 HA	Expression of Codon-optimized Mus SERINC2	C-Terminal HA Tag	This study
11.	pcDNA 3.1(-) Equus SERINC2 HA	Expression of Codon-optimized Equus SERINC2	C-Terminal HA Tag	This study
12.	pcDNA 3.1(-) Yeast TMS-1 HA	Expression of Codon-optimized Yeast TMS-1	C-Terminal HA Tag	This study
13.	pcDNA 3.1(-) Drosophila TMS-1 HA	Expression of Codon-optimized Drosophila TMS-1	C-Terminal HA Tag	This study
14.	pcDNA 3.1(-) Xenopus SERINC5 HA	Expression of Codon-optimized Xenopus SERINC5	C-Terminal HA Tag	This study
15.	pcDNA 3.1(-) Gallus SERINC5 HA	Expression of Codon-optimized Gallus SERINC5	C-Terminal HA Tag	This study
16.	pcDNA 3.1(-) Mus SERINC5 HA	Expression of Codon-optimized Mus SERINC5	C-Terminal HA Tag	This study

17.	pcDNA 3.1(-) Equus SERINC5 HA	Expression of Codon-optimized Equus SERINC5	C-Terminal HA Tag	[2]Chande et al 2016
18.	pcDNA 3.1(-) SERINC3 HA	Expression of Human SERINC3	C-Terminal HA Tag	[3] Rosa et al 2015
19.	pcDNA 3.1(-) SERINC5 HA	Expression of Human SERINC5	C-Terminal HA Tag	[3]Rosa et al 2015
20.	PBJ6 SERINC5 HA	Expression of Human SERINC5 with PBJ6 (weak) promoter	C-Terminal HA Tag	[3] Rosa et al 2015
21.	PBJ5 SIV MAC239 FLAG	Expression of SIV Nef MAC239	C-Terminal FLAG Tag	[3]Rosa et al 2015
22.	PBJ5 Nef Clade C HA	Expression of HIV-1 clade C Nef	C-Terminal HA Tag	[3]Rosa et al 2015
23.	PBJ5 Nef Lai HA	Expression of HIV-1 Lai Nef	C-Terminal HA Tag	[3]Rosa et al 2015
24.	PBJ5 JR-FL	Expression of HIV-1 clade B Env JR-FL		[3]Rosa et al 2015
25.	pcDNA 3.1 (-) ZM109F.PB4	Expression of HIV-1 clade C Env ZM109F		[3]Rosa et al 2015
26.	pCIES	Expression of Foamy virus (FV) Env glycoprotein for production of FV		[4]Trobridge et al 2002 #Addgene - 60010
27.	pCIPS	Expression of FV Pol for production of FV		[4]Trobridgeet al 2002 #Addgene- 60011
28.	pCIGS	Expression of FV Gag for production of FV		[5]Josephson et al 2004 #Addgene- 108312
29.	pΔΦ	Transfer vector for the production of FV		[4]Trobridge et al 2002
30.	pmD2.G	Expression of Vesiculostomatitis virus glycoprotein (VSV-G)		#Addgene- 12259
31.	pcDNA 3.1(-) MLV-glycoGAG HA	Expression of MLV glycoGAG	C-Terminal HA Tag	[6]Pizzato 2010
32.	PHXB2 SRαΔCt	Expression of HIV-1 HXB2 envelope with deletion in C-terminal		[7]Mammano et al 1995
33.	PBJ6 coelacanth SERINC2 HA	Expression of Codon-optimized Coelacanth SERINC2 from a weak promoter	C-Terminal HA Tag	This study
34.	PBJ5 coelacanth SERINC2 HA	Expression of Codon-optimized Coelacanth SERINC2 from a SRα promoter	C-Terminal HA Tag	This study
35.	PBJ6 SERINC1 CO HA	Expression of Codon-optimized Human SERINC1	C-Terminal HA Tag	This study

36.	PBJ5 SERINC1 CO HA	Expression of Codon-optimized Human SERINC1	C-Terminal HA Tag	This study
37.	PBJ6 SERINC2 HA	Expression of Human SERINC2	C-Terminal HA Tag	This study
38.	PBJ5 SERINC2 HA	Expression of Human SERINC2	C-Terminal HA Tag	This study
39.	PBJ6 SERINC3 HA	Expression of Human SERINC3	C-Terminal HA Tag	This study
40.	PBJ5 SERINC3 HA	Expression of Human SERINC3	C-Terminal HA Tag	This study
41.	PBJ6 SERINC4 CO HA	Expression of Codon-optimized Human SERINC4	C-Terminal HA Tag	This study
42.	PBJ5 SERINC4 CO HA	Expression of Codon-optimized Human SERINC4	C-Terminal HA Tag	This study
43.	PBJ5 SERINC5 HA	Expression of Human SERINC5	C-Terminal HA Tag	[3]Rosa et al 2015
44.	pcDNA 3.1(-) SERINC5 HA	Expression of Human SERINC5	C-Terminal HA Tag	[3]Rosa et al 2015

SI Table II: List of Reagents used in this study

Reagent	Company	Catalog No.
Dulbecco's Modified Eagle Medium (DMEM)	Biowest, USA	L0102-500<br>Lot. No. S18531L0102
RPMI 1640	Biowest, USA	L0500-500<br>Lot No. S17846L0500
Fetal Bovine Serum (FBS), Certified, Performance tested. Origin: United states	Gibco, USA	10082-147<br>Lot No. 2097440
L-Glutamine 200mM	Gibco, USA	25030-081
Pen-Strep (Penicillin Streptomycin)	Gibco, USA	15140-122
Hoechst 33342	Sigma Aldrich	14540-100G
OptiMEM	Gibco, USA	S18531L0102
PBS	HyClone, USA	SH30256.02
Poly-L-Lysine	Sigma Aldrich	P4832- 50ML
Paraformaldehyde	Sigma Aldrich	F1635-25ML
BD Perm/wash™	BD Biosciences	51-2091KZ
ProLong™ Glass Antifade Mountant	Molecular Probes	P36982
EMPARTA ACS Sucrose	Merck	1.94921.1021
Sodium dodecyl sulphate	Sigma Aldrich	74255-250G
Trizma® Chloride	Sigma Aldrich	93363-500G
Trizma® Base	Sigma Aldrich	T6066-5KG<br>SLBQ2272V
Bromophenol blue	Sigma Aldrich	B8026-5G<br>MKBX9084V
Glycerol	Sigma Aldrich	G5516-500ml<br>SHBH8759
Tris(2-carboxyethyl) phosphine hydrochloride (TCEP)	Sigma Aldrich	75259
Sodium Chloride	VWR Life Science	0241-1KG
HEPES	VWR Life Science	051-250G
n-Dodecyl-β-D-Maltoside	Thermo Fischer	89903
2xcOmplete™, EDTA-free Protease inhibitor cocktail	Sigma Aldrich	11873580001
PVDF membrane	Immobilon-FL, Merck-Millipore	IPFL00010<br>R7DA8781C
Tricine	MP Biomedicals	103112<br>QR15009
SureCast™ Acrylamide solution (40%)	Invitrogen	HC2040
SureCast™ APS	Invitrogen	HC2005<br>QH205276
SureCast™ TEMED	Invitrogen	HC2002<br>161275304
OdysseyBlockingBuffer	Li-Cor	P/N 927-50003
Tween-20®	Sigma Aldrich	P2287-500ML
Bafilomycin A1	Santa Cruz	SC20155OA

Purified anti-HA.11 Epitope Tag antibody	BioLegend	901501
Beta-Actin Rabbit Monoclonal Antibody	LI-COR Biosciences	926-42210
Goat anti-Mouse IgG Secondary Antibody, Alexa Fluor 488	Thermo Fisher Scientific	A-11001
IRDye 680RD Goat anti-Mouse IgG antibody	LI-COR Biosciences	925-68070
IRDye 800CW Goat anti-Rabbit IgG antibody	LI-COR Biosciences	925-32211
Anti-HIV-1 P24 antibody	NIBSC, UK	N/A
Anti-HIV-1 gp41 Chessie8 antibody	NIH ARP, 13049	

SI Table III: List of primate species along with the corresponding genome assemblies that were corrected using referee pipeline. The raw read data used for genome correction was downloaded from the Short Read Archive (SRA). The Sample accession IDs and run accession IDs for each dataset are provided along with their coverage of the genome assembly.

Serial no.	SPECIES	GENOME	SAMPLE	SRA ACCESSION NO.	COVERAGE	CORRECTED (GENOME)	% ERROR CORRECTED (GENOME)	CORRECTED (EXONIC)	% ERROR CORRECTED (EXONIC)
1	*Saimiri boliviensis boliviensis*	SaiBol1.0	SAMN00672664	SRR315556	9.98	219768	0.008	4616	0.008
				SRR315560	10.19				
				SRR315565	9.87				
				SRR315566	9.39				
			**Total**		39.43				
2	*Papioanubis*	Panu_3.0	SAMN12703524	SRR10076357	115.13	38,95,295	0.132	33835	0.075
3	*Pan paniscus*	panpan1.1	SAMN01920511	SRR740822	5.70	2578182	0.078	171444	0.303
				SRR740823	5.25				

				SRR740824	6.28				
				SRR740825	5.90				
				SRR740827	3.04				
				SRR740828	3.54				
			**Total**		29.71				
4	*Pan troglodytes*	Pan_tro_3.0	SAMEA4557838	ERR1759393	11.38	1,67,190	0.005	1528	0.002
				ERR1759396	12.01				
				ERR1759397	17.90				
				ERR1759398	17.95				
				ERR1759399	19.73				
				ERR1759400	19.63				
			**Total**		98.60				
5	*Rhinopithecusroxellana*	Rrox_v1	SAMN11039125	SRR8822480	48.78	18,61,299	0.065	23295	0.041
				SRR8822482	48.66				
			**Total**		97.44				
6	*Aotusnancymaae*	Anan_2.0	SAMN08978243	SRR7608126	46.22	34,07,700	0.127	35533	0.073
			SAMN08978246	SRR7608129	46.15				
			**Total**		92.37				
7	*Cebus capucinus imitator*	Cebus_imitator-1.0	SAMN04274558	SRR3136934	13.65	14,32,193	0.053	20612	0.036
				SRR3136940	14.73				
				SRR3136953	15.37				
				SRR3136956	13.99				
			**Total**		57.74				
8	*Cercocebusatys*	Caty_1.0	SAMN03121017	SRR1685402	13.69	15,25,253	0.054	13686	0.021

			SAMN03121017	SRR1685403	3.94				
			SAMN03121017	SRR1685404	10.53				
			SAMN03121017	SRR1685405	12.76				
			SAMN03121017	SRR1685406	16.15				
			SAMN03121017	SRR1685407	11.11				
			SAMN03121017	SRR1685408	11.26				
			SAMN03121017	SRR1685409	19.79				
			SAMN03121017	SRR1685410	11.99				
			**Total**		111.21				
9	*Callithrix jacchus*	ASM275486v1	SAMN08957129	SRR7050659	39.61	27,09,429	0.095	32744	0.062
				SRR7050660	63.72				
				SRR7050661	63.72				
			**Total**		167.05				
10	*Chlorocebussabeus*	ChlSab1.1	SAMD00016802	DRR018828	43.10	47,67,051	0.172	47449	0.093
				DRR018829	50.72				
				DRR018830	2.42				
				DRR018831	1.81				
			**Total**		98.05				
11	*Macaca fascicularis*	Macaca_fascicularis_5.0	SAMN06130147	SRR5096603	73.12	56,10,508	0.190	78413	0.125
12		Mleu.le_1.0		SRR1691812	11.58	1,27,163	0.004	1477	0.003

	Mandrillusleucophaeus		SAMN03121813	SRR1691813	15.71				
				SRR1691817	9.25				
				SRR1691820	14.40				
			**Total**		50.94				
13	*Macaca mulatta*	Mmul_10	SAMN10721089	SRR9851966	54.55	53,86,421	0.181	101174	0.128
14	*Homo sapiens*	GRCh38	SAMN11771874	SRR9091899	49.93	17,63,194	0.057	64741	0.044
15	*Gorilla gorilla*	gorGor4	SAMEA5204238	ERR3078030	60.79	1770702	0.058	18426	0.034
			SAMN01920489	SRR748076	7.62				
				SRR748077	6.83				
				SRR748078	8.25				
				SRR748079	8.26				
				SRR748080	7.44				
			**Total**		99.185				

SI Table IV: Ensembl ID's of genes used for the identification of arms-race signatures in primate species.

AIM2	ENSCSAP00000015597	*Chlorocebussabaeus*
	ENSMMUP00000005989	*Macaca mulatta*
	ENSRROP00000018966	*Rhinopithecusroxellana*
	ENSMLEP00000023966	*Mandrillusleucophaeus*
	ENSCATP00000023057	*Cercocebusatys*
	ENSSBOP00000009157	*Saimiriboliviensisboliviensis*
	ENSANAP00000008624	*Aotusnancymaae*
	ENSCCAP00000002426	*Cebuscapucinus imitator*
	ENSMFAP00000017401	*Macacafascicularis*
	ENSPPAP00000027408	*Pan paniscus*
	ENSPANP00000005179	*Papioanubis*
	ENSPTRP00000051692	*Pan troglodytes*
	ENSGGOP00000006002	*Gorilla gorillagorilla*
	ENSCJAP00000011655	*Callithrix jacchus*
	ENSP00000357112	*Homo sapiens*
AVPR1A	ENSCSAP00000000846	*Chlorocebussabaeus*
	ENSMMUP00000000738	*Macaca mulatta*
	ENSMLEP00000010946	*Mandrillusleucophaeus*
	ENSRROP00000030004	*Rhinopithecusroxellana*
	ENSCATP00000040607	*Cercocebusatys*
	ENSMFAP00000034938	*Macacafascicularis*
	ENSPTRP00000061123	*Pan troglodytes*
	ENSPPAP00000007290	*Pan paniscus*
	ENSANAP00000026583	*Aotusnancymaae*
	ENSCCAP00000010825	*Cebuscapucinus imitator*
	ENSSBOP00000024663	*Saimiriboliviensisboliviensis*
	ENSPANP00000010884	*Papioanubis*
	ENSGGOP00000043757	*Gorilla gorillagorilla*
	ENSCJAP00000011245	*Callithrix jacchus*
	ENSP00000299178	*Homo sapiens*
BST2	ENSCSAP00000001914	*Chlorocebussabaeus*
	ENSMMUP00000007685	*Macaca mulatta*
	ENSCATP00000039417	*Cercocebusatys*
	ENSANAP00000020174	*Aotusnancymaae*
	ENSMFAP00000006050	*Macacafascicularis*
	ENSSBOP00000020985	*Saimiriboliviensisboliviensis*
	ENSPANP00000007335	*Papioanubis*
	ENSCCAP00000002697	*Cebuscapucinus imitator*
	ENSMLEP00000030374	*Mandrillusleucophaeus*
	ENSPTRP00000018209	*Pan troglodytes*
	ENSPPAP00000023798	*Pan paniscus*
	ENSRROP00000036864	*Rhinopithecusroxellana*
	ENSGGOP00000014904	*Gorilla gorillagorilla*
	ENSCJAP00000017903	*Callithrix jacchus*
	ENSP00000252593	*Homo sapiens*
CGAS	ENSCSAP00000010160	*Chlorocebussabaeus*

	ENSMMUP00000046784	*Macaca mulatta*
	ENSMFAP00000040312	*Macacafascicularis*
	ENSPPAP00000010871	*Pan paniscus*
	ENSPTRP00000054197	*Pan troglodytes*
	ENSCCAP00000013900	*Cebuscapucinus imitator*
	ENSANAP00000006030	*Aotusnancymaae*
	ENSSBOP00000039530	*Saimiriboliviensisboliviensis*
	ENSCATP00000001541	*Cercocebusatys*
	ENSPANP00000016002	*Papioanubis*
	ENSRROP00000036333	*Rhinopithecusroxellana*
	ENSGGOP00000025666	*Gorilla gorillagorilla*
	ENSCJAP00000018365	*Callithrix jacchus*
	ENSP00000359339	*Homo sapiens*
DDX58	ENSCSAP00000006076	*Chlorocebussabaeus*
	ENSMMUP00000016338	*Macaca mulatta*
	ENSCCAP00000037791	*Cebuscapucinus imitator*
	ENSCATP00000034684	*Cercocebusatys*
	ENSSBOP00000028508	*Saimiriboliviensisboliviensis*
	ENSPPAP00000038038	*Pan paniscus*
	ENSPTRP00000035647	*Pan troglodytes*
	ENSMLEP00000011621	*Mandrillusleucophaeus*
	ENSPANP00000019164	*Aotusnancymaae*
	ENSRROP00000010203	*Rhinopithecusroxellana*
	ENSGGOP00000024163	*Gorilla gorillagorilla*
	ENSCJAP00000014513	*Callithrix jacchus*
	ENSP00000369213	*Homo sapiens*
DHX58	ENSCSAP00000014957	*Chlorocebussabaeus*
	ENSMMUP00000058143	*Macaca mulatta*
	ENSCATP00000041142	*Cercocebusatys*
	ENSMLEP00000037005	*Mandrillusleucophaeus*
	ENSMFAP00000036359	*Macacafascicularis*
	ENSCCAP00000009608	*Cebuscapucinus imitator*
	ENSPPAP00000015998	*Pan paniscus*
	ENSSBOP00000007266	*Saimiriboliviensisboliviensis*
	ENSPANP00000020691	*Papioanubis*
	ENSANAP00000022610	*Aotusnancymaae*
	ENSPTRP00000015656	*Pan troglodytes*
	ENSGGOP00000005347	*Gorilla gorillagorilla*
	ENSCJAP00000051323	*Callithrix jacchus*
	ENSP00000251642	*Homo sapiens*
EIF2AK2	ENSCSAP00000008247	*Chlorocebussabaeus*
	ENSMMUP00000050019	*Macaca mulatta*
	ENSSBOP00000020278	*Saimiriboliviensisboliviensis*
	ENSPPAP00000023149	*Pan paniscus*
	ENSCCAP00000040019	*Cebuscapucinus imitator*
	ENSPANP00000012304	*Papioanubis*

	ENSANAP00000035603	*Aotusnancymaae*
	ENSCATP00000026661	*Cercocebusatys*
	ENSPTRP00000065741	*Pan troglodytes*
	ENSMLEP00000018687	*Mandrillusleucophaeus*
	ENSMFAP00000014830	*Macacafascicularis*
	ENSRROP00000033054	*Rhinopithecusroxellana*
	ENSGGOP00000019160	*Gorilla gorillagorilla*
	ENSCJAP00000006979	*Callithrix jacchus*
	ENSP00000233057	*Homo sapiens*
FITM2	ENSCSAP00000010251	*Chlorocebussabaeus*
	ENSMMUP00000046045	*Macaca mulatta*
	ENSPPAP00000025559	*Pan paniscus*
	ENSMFAP00000026027	*Macacafascicularis*
	ENSSBOP00000004978	*Saimiriboliviensisboliviensis*
	ENSCCAP00000025242	*Cebuscapucinus imitator*
	ENSANAP00000012931	*Aotusnancymaae*
	ENSRROP00000037875	*Rhinopithecusroxellana*
	ENSPANP00000017694	*Papioanubis*
	ENSCATP00000043630	*Cercocebusatys*
	ENSPTRP00000023218	*Pan troglodytes*
	ENSGGOP00000031207	*Gorilla gorillagorilla*
	ENSCJAP00000032798	*Callithrix jacchus*
	ENSP00000380037	*Homo sapiens*
FOXP2	ENSCSAP00000007173	*Chlorocebussabaeus*
	ENSMMUP00000054008	*Macaca mulatta*
	ENSANAP00000012918	*Aotusnancymaae*
	ENSSBOP00000025640	*Saimiriboliviensisboliviensis*
	ENSMLEP00000019003	*Mandrillusleucophaeus*
	ENSCCAP00000000996	*Cebuscapucinus imitator*
	ENSPTRP00000071606	*Pan troglodytes*
	ENSMFAP00000045997	*Macacafascicularis*
	ENSPPAP00000015762	*Pan paniscus*
	ENSRROP00000020401	*Rhinopithecusroxellana*
	ENSGGOP00000047838	*Gorilla gorillagorilla*
	ENSCJAP00000031433	*Callithrix jacchus*
	ENSP00000386200	*Homo sapiens*
GBP5	ENSSBOP00000002096	*Saimiriboliviensisboliviensis*
	ENSANAP00000036403	*Aotusnancymaae*
	ENSCCAP00000002587	*Cebuscapucinus imitator*
	ENSPPAP00000030445	*Pan paniscus*
	ENSPTRP00000001620	*Pan troglodytes*
	ENSGGOP00000001639	*Gorilla gorillagorilla*
	ENSCJAP00000003579	*Callithrix jacchus*
	ENSP00000359488	*Homo sapiens*
IFI16	ENSCSAP00000015600	*Chlorocebussabaeus*
	ENSMMUP00000005984	*Macaca mulatta*

	ENSRROP00000016325	*Rhinopithecusroxellana*
	ENSMLEP00000020240	*Mandrillusleucophaeus*
	ENSCATP00000015644	*Cercocebusatys*
	ENSSBOP00000018169	*Saimiriboliviensisboliviensis*
	ENSANAP00000016439	*Aotusnancymaae*
	ENSCCAP00000024096	*Cebuscapucinus imitator*
	ENSMFAP00000029096	*Macacafascicularis*
	ENSPPAP00000013418	*Pan paniscus*
	ENSPANP00000011185	*Papioanubis*
	ENSPTRP00000065596	*Pan troglodytes*
	ENSGGOP00000005994	*Gorilla gorillagorilla*
	ENSCJAP00000048962	*Callithrix jacchus*
	ENSP00000357113	*Homo sapiens*
IFIH1	ENSCSAP00000011653	*Chlorocebussabaeus*
	ENSMMUP00000004277	*Macaca mulatta*
	ENSCCAP00000018819	*Cebuscapucinus imitator*
	ENSPPAP00000031468	*Pan paniscus*
	ENSRROP00000013435	*Rhinopithecusroxellana*
	ENSSBOP00000019200	*Saimiriboliviensisboliviensis*
	ENSANAP00000020745	*Aotusnancymaae*
	ENSCATP00000016245	*Cercocebusatys*
	ENSMFAP00000034733	*Macacafascicularis*
	ENSMLEP00000000093	*Mandrillusleucophaeus*
	ENSPTRP00000021498	*Pan troglodytes*
	ENSGGOP00000015505	*Gorilla gorillagorilla*
	ENSCJAP00000011007	*Callithrix jacchus*
	ENSP00000497271	*Homo sapiens*
IFITM1	ENSCSAP00000007315	*Chlorocebussabaeus*
	ENSMLEP00000021135	*Macaca mulatta*
	ENSCATP00000010879	*Cercocebusatys*
	ENSPPAP00000020303	*Pan paniscus*
	ENSMFAP00000006911	*Macacafascicularis*
	ENSPTRP00000048340	*Pan troglodytes*
	ENSGGOP00000003322	*Gorilla gorillagorilla*
	ENSP00000330825	*Homo sapiens*
IFITM5	ENSCSAP00000007320	*Chlorocebussabaeus*
	ENSMMUP00000006155	*Macaca mulatta*
	ENSCCAP00000010892	*Cebuscapucinus imitator*
	ENSCATP00000014546	*Cercocebusatys*
	ENSPPAP00000021935	*Pan paniscus*
	ENSMFAP00000026179	*Macacafascicularis*
	ENSPANP00000010044	*Papioanubis*
	ENSANAP00000034272	*Aotusnancymaae*
	ENSRROP00000004481	*Rhinopithecusroxellana*
	ENSSBOP00000002681	*Saimiriboliviensisboliviensis*
	ENSPTRP00000048341	*Pan troglodytes*

	ENSCJAP00000058286	*Callithrix jacchus*
	ENSP00000372059	*Homo sapiens*
IZUMO1	ENSCSAP00000015233	*Chlorocebussabaeus*
	ENSMMUP00000005363	*Macaca mulatta*
	ENSMFAP00000011540	*Macacafascicularis*
	ENSANAP00000019391	*Aotusnancymaae*
	ENSPANP00000001077	*Papioanubis*
	ENSRROP00000002714	*Rhinopithecusroxellana*
	ENSMLEP00000038185	*Mandrillusleucophaeus*
	ENSSBOP00000012431	*Saimiriboliviensisboliviensis*
	ENSCATP00000028216	*Cercocebusatys*
	ENSCCAP00000027546	*Cebuscapucinus imitator*
	ENSPTRP00000019303	*Pan troglodytes*
	ENSPPAP00000010843	*Pan paniscus*
	ENSGGOP00000001225	*Gorilla gorillagorilla*
	ENSCJAP00000000528	*Callithrix jacchus*
	ENSP00000327786	*Homo sapiens*
IZUMO4	ENSCSAP00000008055	*Chlorocebussabaeus*
	ENSMMUP00000031995	*Macaca mulatta*
	ENSSBOP00000022676	*Saimiriboliviensisboliviensis*
	ENSMFAP00000025065	*Macacafascicularis*
	ENSCATP00000028372	*Cercocebusatys*
	ENSMLEP00000016970	*Mandrillusleucophaeus*
	ENSPANP00000000628	*Papioanubis*
	ENSPTRP00000093295	*Pan troglodytes*
	ENSANAP00000026438	*Aotusnancymaae*
	ENSCCAP00000005740	*Cebuscapucinus imitator*
	ENSPPAP00000038878	*Pan paniscus*
	ENSRROP00000037849	*Rhinopithecusroxellana*
	ENSGGOP00000029568	*Gorilla gorillagorilla*
	ENSCJAP00000060932	*Callithrix jacchus*
	ENSP00000378712	*Homo sapiens*
LYZ	ENSCSAP00000000581	*Chlorocebussabaeus*
	ENSMMUP00000047515	*Macaca mulatta*
	ENSRROP00000019722	*Rhinopithecusroxellana*
	ENSMFAP00000007753	*Macacafascicularis*
	ENSPTRP00000050073	*Pan troglodytes*
	ENSPPAP00000032089	*Pan paniscus*
	ENSCCAP00000031439	*Cebuscapucinus imitator*
	ENSSBOP00000001890	*Saimiriboliviensisboliviensis*
	ENSMLEP00000013069	*Mandrillusleucophaeus*
	ENSCATP00000036951	*Cercocebusatys*
	ENSANAP00000002950	*Aotusnancymaae*
	ENSPANP00000012261	*Papioanubis*
	ENSGGOP00000010835	*Gorilla gorillagorilla*
	ENSCJAP00000003201	*Callithrix jacchus*

	ENSP00000261267	*Homo sapiens*
MARCH8	ENSMMUP00000017652	*Macaca mulatta*
	ENSCCAP00000021378	*Cebuscapucinus imitator*
	ENSSBOP00000014336	*Saimiriboliviensisboliviensis*
	ENSCATP00000005935	*Cercocebusatys*
	ENSPPAP00000010391	*Pan paniscus*
	ENSANAP00000028626	*Aotusnancymaae*
	ENSMFAP00000032591	*Macacafascicularis*
	ENSMLEP00000005895	*Mandrillusleucophaeus*
	ENSRROP00000026406	*Rhinopithecusroxellana*
	ENSPTRP00000075641	*Pan troglodytes*
	ENSGGOP00000014630	*Gorilla gorillagorilla*
	ENSCJAP00000049902	*Callithrix jacchus*
	ENSP00000411848	*Homo sapiens*
MNDA	ENSCSAP00000015605	*Chlorocebussabaeus*
	ENSMMUP00000024276	*Macaca mulatta*
	ENSRROP00000017369	*Rhinopithecusroxellana*
	ENSCATP00000015962	*Cercocebusatys*
	ENSMLEP00000008528	*Mandrillusleucophaeus*
	ENSANAP00000025366	*Aotusnancymaae*
	ENSCCAP00000031321	*Cebuscapucinus imitator*
	ENSMFAP00000020850	*Macacafascicularis*
	ENSPPAP00000019524	*Pan paniscus*
	ENSPANP00000011116	*Papioanubis*
	ENSPTRP00000089964	*Pan troglodytes*
	ENSGGOP00000005810	*Gorilla gorillagorilla*
	ENSP00000357123	*Homo sapiens*
MOV10	ENSCSAP00000016427	*Chlorocebussabaeus*
	ENSCATP00000026645	*Cercocebusatys*
	ENSCCAP00000033856	*Cebuscapucinus imitator*
	ENSMLEP00000000623	*Mandrillusleucophaeus*
	ENSRROP00000035282	*Rhinopithecusroxellana*
	ENSANAP00000033562	*Aotusnancymaae*
	ENSMFAP00000005215	*Macacafascicularis*
	ENSPPAP00000015996	*Pan paniscus*
	ENSSBOP00000001141	*Saimiriboliviensisboliviensis*
	ENSPANP00000018271	*Papioanubis*
	ENSPTRP00000001914	*Pan troglodytes*
	ENSGGOP00000044987	*Gorilla gorillagorilla*
	ENSCJAP00000025111	*Callithrix jacchus*
	ENSP00000399797	*Homo sapiens*
OASL	ENSCSAP00000015814	*Chlorocebussabaeus*
	ENSMMUP00000049271	*Macaca mulatta*
	ENSRROP00000024149	*Rhinopithecusroxellana*
	ENSMFAP00000023209	*Macacafascicularis*
	ENSPTRP00000009423	*Pan troglodytes*

	ENSPPAP00000025905	*Pan paniscus*
	ENSCCAP00000020404	*Cebuscapucinus imitator*
	ENSCATP00000022480	*Cercocebusatys*
	ENSSBOP00000039064	*Saimiriboliviensisboliviensis*
	ENSPANP00000011169	*Papioanubis*
	ENSMLEP00000027551	*Mandrillusleucophaeus*
	ENSGGOP00000011828	*Gorilla gorillagorilla*
	ENSCJAP00000062958	*Callithrix jacchus*
	ENSP00000257570	*Homo sapiens*
OXTR	ENSCSAP00000007016	*Chlorocebussabaeus*
	ENSMMUP00000012694	*Macaca mulatta*
	ENSMFAP00000002720	*Macacafascicularis*
	ENSANAP00000027621	*Aotusnancymaae*
	ENSPANP00000002118	*Papioanubis*
	ENSCATP00000036495	*Cercocebusatys*
	ENSSBOP00000031060	*Saimiriboliviensisboliviensis*
	ENSPTRP00000025138	*Pan troglodytes*
	ENSCCAP00000036233	*Cebuscapucinus imitator*
	ENSMLEP00000000363	*Mandrillusleucophaeus*
	ENSRROP00000038241	*Rhinopithecusroxellana*
	ENSGGOP00000023689	*Gorilla gorillagorilla*
	ENSCJAP00000029299	*Callithrix jacchus*
	ENSP00000324270	*Homo sapiens*
PARP4	ENSCSAP00000014024	*Chlorocebussabaeus*
	ENSMMUP00000055967	*Macaca mulatta*
	ENSANAP00000006334	*Aotusnancymaae*
	ENSRROP00000033710	*Rhinopithecusroxellana*
	ENSPTRP00000009733	*Pan troglodytes*
	ENSCATP00000024537	*Cercocebusatys*
	ENSCCAP00000029652	*Cebuscapucinus imitator*
	ENSPPAP00000006029	*Pan paniscus*
	ENSMLEP00000033757	*Mandrillusleucophaeus*
	ENSPANP00000008644	*Papioanubis*
	ENSGGOP00000002899	*Gorilla gorillagorilla*
	ENSCJAP00000008182	*Callithrix jacchus*
	ENSP00000371419	*Homo sapiens*
PIGR	ENSCSAP00000011135	*Chlorocebussabaeus*
	ENSMMUP00000014705	*Macaca mulatta*
	ENSMLEP00000011088	*Mandrillusleucophaeus*
	ENSANAP00000017507	*Aotusnancymaae*
	ENSMFAP00000038512	*Macacafascicularis*
	ENSRROP00000027006	*Rhinopithecusroxellana*
	ENSCCAP00000026510	*Chlorocebussabaeus*
	ENSSBOP00000039951	*Saimiriboliviensisboliviensis*
	ENSPPAP00000025593	*Pan paniscus*
	ENSCATP00000007081	*Cercocebusatys*

	ENSPANP00000004132	*Papioanubis*
	ENSPTRP00000046994	*Pan troglodytes*
	ENSGGOP00000009769	*Gorilla gorillagorilla*
	ENSCJAP00000034289	*Callithrix jacchus*
	ENSP00000348888	*Homo sapiens*
PYHIN1	ENSCSAP00000015604	*Chlorocebussabaeus*
	ENSMMUP00000005981	*Macaca mulatta*
	ENSRROP00000021042	*Rhinopithecusroxellana*
	ENSMLEP00000017665	*Mandrillusleucophaeus*
	ENSCATP00000012401	*Cercocebusatys*
	ENSSBOP00000031878	*Saimiriboliviensisboliviensis*
	ENSANAP00000041854	*Aotusnancymaae*
	ENSCCAP00000022793	*Cebuscapucinus imitator*
	ENSMFAP00000009323	*Macacafascicularis*
	ENSPPAP00000034760	*Pan paniscus*
	ENSPANP00000007104	*Papioanubis*
	ENSPTRP00000002552	*Pan troglodytes*
	ENSGGOP00000012088	*Gorilla gorillagorilla*
	ENSCJAP00000045837	*Callithrix jacchus*
	ENSP00000357122	*Homo sapiens*
RHO	ENSCSAP00000005330	*Chlorocebussabaeus*
	ENSMFAP00000031097	*Macacafascicularis*
	ENSRROP00000019551	*Rhinopithecusroxellana*
	ENSMLEP00000035242	*Mandrillusleucophaeus*
	ENSPPAP00000036980	*Pan paniscus*
	ENSANAP00000030374	*Aotusnancymaae*
	ENSCATP00000030887	*Cercocebusatys*
	ENSSBOP00000007576	*Saimiriboliviensisboliviensis*
	ENSCCAP00000000656	*Cebuscapucinus imitator*
	ENSPTRP00000026495	*Pan troglodytes*
	ENSPANP00000014886	*Papioanubis*
	ENSGGOP00000010459	*Gorilla gorillagorilla*
	ENSCJAP00000031629	*Callithrix jacchus*
	ENSP00000296271	*Homo sapiens*
RNASEL	ENSCSAP00000008696	*Chlorocebussabaeus*
	ENSRROP00000024343	*Rhinopithecusroxellana*
	ENSMLEP00000026344	*Mandrillusleucophaeus*
	ENSSBOP00000036914	*Saimiriboliviensisboliviensis*
	ENSANAP00000037085	*Aotusnancymaae*
	ENSCCAP00000028953	*Cebuscapucinus imitator*
	ENSMFAP00000027096	*Macacafascicularis*
	ENSPPAP00000012730	*Pan paniscus*
	ENSCATP00000031362	*Cercocebusatys*
	ENSPANP00000010115	*Papioanubis*
	ENSPTRP00000002931	*Pan troglodytes*
	ENSGGOP00000019722	*Gorilla gorillagorilla*

	ENSCJAP00000047869	*Callithrix jacchus*
	ENSP00000356530	*Homo sapiens*
RSAD2	ENSCSAP00000003326	*Chlorocebussabaeus*
	ENSMMUP00000048848	*Macaca mulatta*
	ENSPPAP00000033245	*Pan paniscus*
	ENSCATP00000011923	*Cercocebusatys*
	ENSCCAP00000028838	*Cebuscapucinus imitator*
	ENSANAP00000029024	*Aotusnancymaae*
	ENSPANP00000012843	*Papioanubis*
	ENSMLEP00000023754	*Mandrillusleucophaeus*
	ENSPTRP00000019995	*Pan troglodytes*
	ENSSBOP00000015080	*Saimiriboliviensisboliviensis*
	ENSRROP00000000609	*Rhinopithecusroxellana*
	ENSMFAP00000020779	*Macacafascicularis*
	ENSGGOP00000010453	*Gorilla gorillagorilla*
	ENSCJAP00000059848	*Callithrix jacchus*
	ENSP00000371471	*Homo sapiens*
SAMHD1	ENSCSAP00000006187	*Chlorocebussabaeus*
	ENSMMUP00000002791	*Macaca mulatta*
	ENSPPAP00000030664	*Pan paniscus*
	ENSMFAP00000007165	*Macacafascicularis*
	ENSSBOP00000003866	*Saimiriboliviensisboliviensis*
	ENSANAP00000012153	*Aotusnancymaae*
	ENSRROP00000021248	*Rhinopithecusroxellana*
	ENSPANP00000015945	*Papioanubis*
	ENSCATP00000006862	*Cercocebusatys*
	ENSMLEP00000025424	*Mandrillusleucophaeus*
	ENSPTRP00000023119	*Pan troglodytes*
	ENSGGOP00000011054	*Gorilla gorillagorilla*
	ENSCJAP00000002207	*Callithrix jacchus*
	ENSP00000493536	*Homo sapiens*
SERINC1	ENSCSAP00000012322	*Chlorocebussabaeus*
	ENSMMUP00000010383	*Macaca mulatta*
	ENSANAP00000006694	*Aotusnancymaae*
	ENSMFAP00000024219	*Macacafascicularis*
	ENSPPAP00000036490	*Pan paniscus*
	ENSPTRP00000031691	*Pan troglodytes*
	ENSMLEP00000010825	*Mandrillusleucophaeus*
	ENSCATP00000033806	*Cercocebusatys*
	ENSCCAP00000017537	*Cebuscapucinus imitator*
	ENSSBOP00000029107	*Saimiriboliviensisboliviensis*
	ENSPANP00000007615	*Papioanubis*
	ENSRROP00000037067	*Rhinopithecusroxellana*
	ENSCJAP00000065527	*Callithrix jacchus*
	ENSP00000342962	*Homo sapiens*
SERINC2	ENSMMUP00000047763	*Macaca mulatta*

	ENSANAP00000016475	*Aotusnancymaae*
	ENSMLEP00000025279	*Mandrillusleucophaeus*
	ENSSBOP00000026414	*Saimiriboliviensisboliviensis*
	ENSCCAP00000010067	*Cebuscapucinus imitator*
	ENSMFAP00000029968	*Macacafascicularis*
	ENSCATP00000043122	*Cercocebusatys*
	ENSPANP00000025152	*Papioanubis*
	ENSRROP00000045284	*Rhinopithecusroxellana*
	ENSPTRP00000069726	*Pan troglodytes*
	ENSGGOP00000008042	*Gorilla gorillagorilla*
	ENSCJAP00000056519	*Callithrix jacchus*
	ENSP00000362814	*Homo sapiens*
SERINC3	ENSMMUP00000018361	*Macaca mulatta*
	ENSMFAP00000027636	*Macacafascicularis*
	ENSSBOP00000033724	*Saimiriboliviensisboliviensis*
	ENSCCAP00000027510	*Cebuscapucinus imitator*
	ENSANAP00000037590	*Aotusnancymaae*
	ENSMLEP00000023632	*Mandrillusleucophaeus*
	ENSRROP00000002722	*Rhinopithecusroxellana*
	ENSPANP00000041110	*Papioanubis*
	ENSCATP00000011331	*Cercocebusatys*
	ENSPTRP00000023229	*Pan troglodytes*
	ENSGGOP00000015025	*Gorilla gorillagorilla*
	ENSCJAP00000044612	*Callithrix jacchus*
	ENSP00000340243	*Homo sapiens*
SERINC4	ENSMMUP00000044014	*Macaca mulatta*
	ENSCATP00000001732	*Cercocebusatys*
	ENSMLEP00000014047	*Mandrillusleucophaeus*
	ENSANAP00000038155	*Aotusnancymaae*
	ENSSBOP00000022789	*Saimiriboliviensisboliviensis*
	ENSMFAP00000006623	*Macacafascicularis*
	ENSPANP00000014686	*Papioanubis*
	ENSCCAP00000016466	*Cebuscapucinus imitator*
	ENSRROP00000030126	*Rhinopithecusroxellana*
	ENSPPAP00000017524	*Pan paniscus*
	ENSPTRP00000011970	*Pan troglodytes*
	ENSGGOP00000014445	*Gorilla gorillagorilla*
	ENSCJAP00000004664	*Callithrix jacchus*
	ENSP00000319796	*Homo sapiens*
SERINC5	ENSCSAP00000011267	*Chlorocebussabaeus*
	ENSMMUP00000053038	*Macaca mulatta*
	ENSPPAP00000034192	*Pan paniscus*
	ENSANAP00000013767	*Aotusnancymaae*
	ENSMFAP00000039686	*Macacafascicularis*
	ENSCATP00000042781	*Cercocebusatys*
	ENSPTRP00000086189	*Pan troglodytes*

	ENSPANP00000000842	*Papioanubis*
	ENSCCAP00000030468	*Cebuscapucinus imitator*
	ENSGGOP00000040571	*Gorilla gorillagorilla*
	ENSP00000426237	*Homo sapiens*
SHH	ENSCSAP00000002769	*Chlorocebussabaeus*
	ENSMMUP00000059872	*Macaca mulatta*
	ENSCATP00000031175	*Cercocebusatys*
	ENSPANP00000017379	*Papioanubis*
	ENSCCAP00000032826	*Cebuscapucinus imitator*
	ENSPTRP00000075730	*Pan troglodytes*
	ENSMFAP00000031777	*Macacafascicularis*
	ENSPPAP00000020557	*Pan paniscus*
	ENSGGOP00000013669	*Gorilla gorillagorilla*
	ENSCJAP00000037954	*Callithrix jacchus*
	ENSP00000297261	*Homo sapiens*
SLFN12	ENSCSAP00000002585	*Chlorocebussabaeus*
	ENSRROP00000004439	*Rhinopithecusroxellana*
	ENSPTRP00000070164	*Pan troglodytes*
	ENSMFAP00000015027	*Macacafascicularis*
	ENSCATP00000028655	*Cercocebusatys*
	ENSMLEP00000014713	*Mandrillusleucophaeus*
	ENSPPAP00000001994	*Pan paniscus*
	ENSPANP00000020042	*Papioanubis*
	ENSPTRP00000090493	*Pan troglodytes*
	ENSGGOP00000001594	*Gorilla gorillagorilla*
	ENSP00000378063	*Homo sapiens*
SLFN13	ENSCSAP00000002579	*Chlorocebussabaeus*
	ENSMMUP00000007336	*Macaca mulatta*
	ENSRROP00000004431	*Rhinopithecusroxellana*
	ENSMFAP00000002369	*Macacafascicularis*
	ENSCATP00000003294	*Cercocebusatys*
	ENSMLEP00000033156	*Mandrillusleucophaeus*
	ENSPPAP00000032337	*Pan paniscus*
	ENSPANP00000045898	*Papioanubis*
	ENSPTRP00000015384	*Pan troglodytes*
	ENSGGOP00000013938	*Gorilla gorillagorilla*
	ENSP00000285013	*Homo sapiens*
SLFN14	ENSCSAP00000002570	*Chlorocebussabaeus*
	ENSMMUP00000043975	*Macaca mulatta*
	ENSRROP00000038689	*Rhinopithecusroxellana*
	ENSANAP00000041665	*Aotusnancymaae*
	ENSMFAP00000037303	*Macacafascicularis*
	ENSSBOP00000022191	*Saimiriboliviensisboliviensis*
	ENSCATP00000007341	*Cercocebusatys*
	ENSCCAP00000014286	*Cebuscapucinus imitator*
	ENSMLEP00000009630	*Mandrillusleucophaeus*

	ENSPPAP00000041083	*Pan paniscus*
	ENSPANP00000020047	*Papioanubis*
	ENSPTRP00000060942	*Pan troglodytes*
	ENSCJAP00000027326	*Callithrix jacchus*
	ENSP00000391101	*Homo sapiens*
SLFN5	ENSCSAP00000002592	*Chlorocebussabaeus*
	ENSMMUP00000013183	*Macaca mulatta*
	ENSANAP00000004820	*Aotusnancymaae*
	ENSMFAP00000035670	*Macacafascicularis*
	ENSSBOP00000010064	*Saimiriboliviensisboliviensis*
	ENSCATP00000039884	*Cercocebusatys*
	ENSCCAP00000031772	*Cebuscapucinus imitator*
	ENSRROP00000016934	*Rhinopithecusroxellana*
	ENSPPAP00000029778	*Pan paniscus*
	ENSMLEP00000019851	*Mandrillusleucophaeus*
	ENSPANP00000020039	*Papioanubis*
	ENSPTRP00000060122	*Pan troglodytes*
	ENSGGOP00000010975	*Gorilla gorillagorilla*
	ENSCJAP00000027305	*Callithrix jacchus*
	ENSP00000299977	*Homo sapiens*
SUN2	ENSCSAP00000002773	*Chlorocebussabaeus*
	ENSMMUP00000052476	*Macaca mulatta*
	ENSCATP00000038305	*Cercocebusatys*
	ENSMFAP00000012163	*Macacafascicularis*
	ENSPTRP00000046802	*Pan troglodytes*
	ENSPPAP00000031471	*Pan paniscus*
	ENSRROP00000029183	*Rhinopithecusroxellana*
	ENSCCAP00000024340	*Cebuscapucinus imitator*
	ENSPANP00000013343	*Papioanubis*
	ENSSBOP00000019372	*Saimiriboliviensisboliviensis*
	ENSMLEP00000017668	*Mandrillusleucophaeus*
	ENSANAP00000012893	*Aotusnancymaae*
	ENSGGOP00000033124	*Gorilla gorillagorilla*
	ENSCJAP00000049139	*Callithrix jacchus*
	ENSP00000385616	*Homo sapiens*
TFRC	ENSCSAP00000004805	*Chlorocebussabaeus*
	ENSMMUP00000002816	*Macaca mulatta*
	ENSCCAP00000025339	*Cebuscapucinus imitator*
	ENSMLEP00000017167	*Mandrillusleucophaeus*
	ENSMFAP00000046017	*Macacafascicularis*
	ENSPPAP00000004959	*Pan paniscus*
	ENSCATP00000022726	*Cercocebusatys*
	ENSANAP00000024811	*Aotusnancymaae*
	ENSPANP00000007350	*Papioanubis*
	ENSPTRP00000046807	*Pan troglodytes*
	ENSSBOP00000037151	*Saimiriboliviensisboliviensis*

	ENSRROP00000008289	*Rhinopithecusroxellana*
	ENSGGOP00000007085	*Gorilla gorillagorilla*
	ENSCJAP00000037296	*Callithrix jacchus*
	ENSP00000376197	*Homo sapiens*
TLR10	ENSMMUP00000035347	*Macaca mulatta*
	ENSMLEP00000000843	*Mandrillusleucophaeus*
	ENSMFAP00000013747	*Macacafascicularis*
	ENSCATP00000006471	*Cercocebusatys*
	ENSPPAP00000003305	*Pan paniscus*
	ENSPANP00000028159	*Papioanubis*
	ENSPTRP00000027515	*Pan troglodytes*
	ENSRROP00000004802	*Rhinopithecusroxellana*
	ENSGGOP00000022546	*Gorilla gorillagorilla*
	ENSCJAP00000001437	*Callithrix jacchus*
	ENSP00000308925	*Homo sapiens*
TLR1	ENSCSAP00000019188	*Chlorocebussabaeus*
	ENSMMUP00000022021	*Macaca mulatta*
	ENSMLEP00000006752	*Mandrillusleucophaeus*
	ENSMFAP00000013794	*Macacafascicularis*
	ENSCATP00000005885	*Cercocebusatys*
	ENSPPAP00000004374	*Pan paniscus*
	ENSPANP00000000454	*Papioanubis*
	ENSCJAP00000001425	*Callithrix jacchus*
	ENSP00000354932	*Homo sapiens*
TLR2	ENSCSAP00000017617	*Chlorocebussabaeus*
	ENSMMUP00000020834	*Macaca mulatta*
	ENSMLEP00000000183	*Mandrillusleucophaeus*
	ENSCATP00000007629	*Cercocebusatys*
	ENSMFAP00000011078	*Macacafascicularis*
	ENSCCAP00000036017	*Cebuscapucinus imitator*
	ENSPPAP00000007123	*Pan paniscus*
	ENSPANP00000010674	*Papioanubis*
	ENSPTRP00000028379	*Pan troglodytes*
	ENSRROP00000006998	*Rhinopithecusroxellana*
	ENSGGOP00000003012	*Gorilla gorillagorilla*
	ENSCJAP00000040512	*Callithrix jacchus*
	ENSP00000260010	*Homo sapiens*
TLR3	ENSCSAP00000015097	*Chlorocebussabaeus*
	ENSMMUP00000028658	*Macaca mulatta*
	ENSANAP00000009875	*Aotusnancymaae*
	ENSRROP00000001660	*Rhinopithecusroxellana*
	ENSMFAP00000040196	*Macacafascicularis*
	ENSSBOP00000025194	*Saimiriboliviensisboliviensis*
	ENSCCAP00000016309	*Cebuscapucinus imitator*
	ENSCATP00000011052	*Cercocebusatys*
	ENSPPAP00000016321	*Pan paniscus*

	ENSMLEP00000028707	*Mandrillusleucophaeus*
	ENSPANP00000002420	*Papioanubis*
	ENSPTRP00000028569	*Pan troglodytes*
	ENSGGOP00000027247	*Gorilla gorillagorilla*
	ENSCJAP00000011150	*Callithrix jacchus*
	ENSP00000296795	*Homo sapiens*
TLR4	ENSCSAP00000009311	*Chlorocebussabaeus*
	ENSMMUP00000011954	*Macaca mulatta*
	ENSCCAP00000005617	*Cebuscapucinus imitator*
	ENSSBOP00000024949	*Saimiriboliviensisboliviensis*
	ENSMLEP00000013603	*Mandrillusleucophaeus*
	ENSANAP00000017232	*Aotusnancymaae*
	ENSMFAP00000043191	*Macacafascicularis*
	ENSCATP00000041789	*Cercocebusatys*
	ENSPPAP00000016171	*Pan paniscus*
	ENSPTRP00000036398	*Pan troglodytes*
	ENSRROP00000017430	*Rhinopithecusroxellana*
	ENSPANP00000029907	*Papioanubis*
	ENSGGOP00000020706	*Gorilla gorillagorilla*
	ENSGGOP00000021036	*Gorilla gorillagorilla*
	ENSCJAP00000052512	*Callithrix jacchus*
	ENSP00000363089	*Homo sapiens*
TLR5	ENSMMUP00000001163	*Macaca mulatta*
	ENSCCAP00000033768	*Cebuscapucinus imitator*
	ENSMFAP00000013602	*Macacafascicularis*
	ENSPPAP00000001949	*Pan paniscus*
	ENSPANP00000014798	*Papioanubis*
	ENSPTRP00000076187	*Pan troglodytes*
	ENSCJAP00000040724	*Callithrix jacchus*
	ENSP00000496355	*Homo sapiens*
TLR6	ENSCSAP00000019187	*Chlorocebussabaeus*
	ENSMMUP00000003242	*Macaca mulatta*
	ENSANAP00000028007	*Aotusnancymaae*
	ENSP00000389600	*Homo sapiens*
TLR7	ENSCSAP00000019028	*Chlorocebussabaeus*
	ENSMMUP00000042318	*Macaca mulatta*
	ENSRROP00000004175	*Rhinopithecusroxellana*
	ENSCCAP00000011708	*Cebuscapucinus imitator*
	ENSPTRP00000047494	*Pan troglodytes*
	ENSANAP00000026575	*Aotusnancymaae*
	ENSPPAP00000009572	*Pan paniscus*
	ENSMLEP00000033999	*Mandrillusleucophaeus*
	ENSSBOP00000003105	*Saimiriboliviensisboliviensis*
	ENSMFAP00000028423	*Macacafascicularis*
	ENSPANP00000008761	*Papioanubis*
	ENSCATP00000042815	*Cercocebusatys*

	ENSGGOP00000020624	*Gorilla gorillagorilla*
	ENSCJAP00000007128	*Callithrix jacchus*
	ENSP00000370034	*Homo sapiens*
TLR8	ENSRROP00000011677	*Rhinopithecusroxellana*
	ENSPTRP00000062546	*Pan troglodytes*
	ENSANAP00000025430	*Aotusnancymaae*
	ENSPPAP00000033784	*Pan paniscus*
	ENSMLEP00000026501	*Mandrillusleucophaeus*
	ENSMFAP00000005531	*Macacafascicularis*
	ENSPANP00000006798	*Papioanubis*
	ENSCATP00000043467	*Cercocebusatys*
	ENSGGOP00000012021	*Gorilla gorillagorilla*
	ENSCJAP00000007141	*Callithrix jacchus*
	ENSP00000312082	*Homo sapiens*
ZBTB7C	ENSCSAP00000014037	*Chlorocebussabaeus*
	ENSPPAP00000034086	*Pan paniscus*
	ENSSBOP00000022834	*Saimiriboliviensisboliviensis*
	ENSANAP00000030740	*Aotusnancymaae*
	ENSMLEP00000021371	*Mandrillusleucophaeus*
	ENSCCAP00000033721	*Cebuscapucinus imitator*
	ENSMFAP00000017042	*Macacafascicularis*
	ENSCATP00000043777	*Cercocebusatys*
	ENSPANP00000010915	*Papioanubis*
	ENSRROP00000006927	*Rhinopithecusroxellana*
	ENSPTRP00000017011	*Pan troglodytes*
	ENSGGOP00000017890	*Gorilla gorillagorilla*
	ENSCJAP00000066208	*Callithrix jacchus*
	ENSP00000468782	*Homo sapiens*

Supplemental references:

1. Sood, C., Marin, M., Chande, A., Pizzato, M. & Melikyan, G. B. SERINC5 protein inhibits HIV-1 fusion pore formation by promoting functional inactivation of envelope glycoproteins. *J. Biol. Chem.* **292**, (2017).
2. Chande, A. *et al.* S2 from equine infectious anemia virus is an infectivity factor which counteracts the retroviral inhibitors SERINC5 and SERINC3. *Proc. Natl. Acad. Sci. U. S. A.* **113**, 13197–13202 (2016).
3. Rosa, A. *et al.* HIV-1 Nef promotes infection by excluding SERINC5 from virion incorporation. *Nature* **526**, 212–217 (2015).
4. Trobridge, G., Josephson, N., Vassilopoulos, G., Mac, J. & Russell, D. W. Improved foamy virus vectors with minimal viral sequences. *Mol. Ther.* **6**, 321–328 (2002).
5. Josephson, N. C., Trobridge, G. & Russell, D. W. Transduction of Long-Term and Mobilized Peripheral Blood-Derived NOD/SCID Repopulating Cells by Foamy Virus Vectors. *Hum. Gene Ther.* **15**, 87–92 (2004).
6. Pizzato, M. MLV glycosylated-gag is an infectivity factor that rescues Nef-deficient HIV-1. *Proc. Natl. Acad. Sci. U. S. A.* **107**, 9364–9369 (2010).
7. Mammano, F., Kondo, E., Sodroski, J., Bukovsky, A. & Gottlinger, H. G. Rescue of human immunodeficiency virus type 1 matrix protein mutants by envelope glycoproteins with short cytoplasmic domains. *J. Virol.* (1995).
8. Tsirigos, K. D., Peters, C., Shu, N., Käll, L. & Elofsson, A. The TOPCONS web server for consensus prediction of membrane protein topology and signal peptides. *Nucleic Acids Res.* **43**, W401–W407 (2015).

Supplemental Material legends

SI Table I: List of Plasmids used in this study

SI Table II: List of Reagents used in this study

SI Table III: List of primate species along with the corresponding genome assemblies that were corrected using referee pipeline. The raw read data used for genome correction was downloaded from the Short Read Archive (SRA). The Sample accession IDs and run accession IDs for each dataset are provided along with their coverage of the genome assembly.

SI Table IV: Ensembl ID's of genes used for the identification of arms-race signatures in primate species.

SI dataset: Gene expression evolution of SERINC genes in vertebrates

SI Video-1: Rotating 3-D view of the CryoEM structure of TMS-1 monomer from D. melanogaster reported in Pye et al 2020 visualized using ChimeraX software. The regions highlighted in the MSA from Fig-7A are colored white (the first region), yellow (the second region), and green (the third region).

SI Video-2: Rotating 3-D view of the CryoEM structure of TMS-1 hexamer from D. melanogaster reported in Pye et al 2020 visualized using ChimeraX software. The regions highlighted in the MSA from Fig-7A are colored white (the first region), yellow (the second region), and green (the third region)

SI dataset: Gene expression evolution of SERINC genes in vertebrates

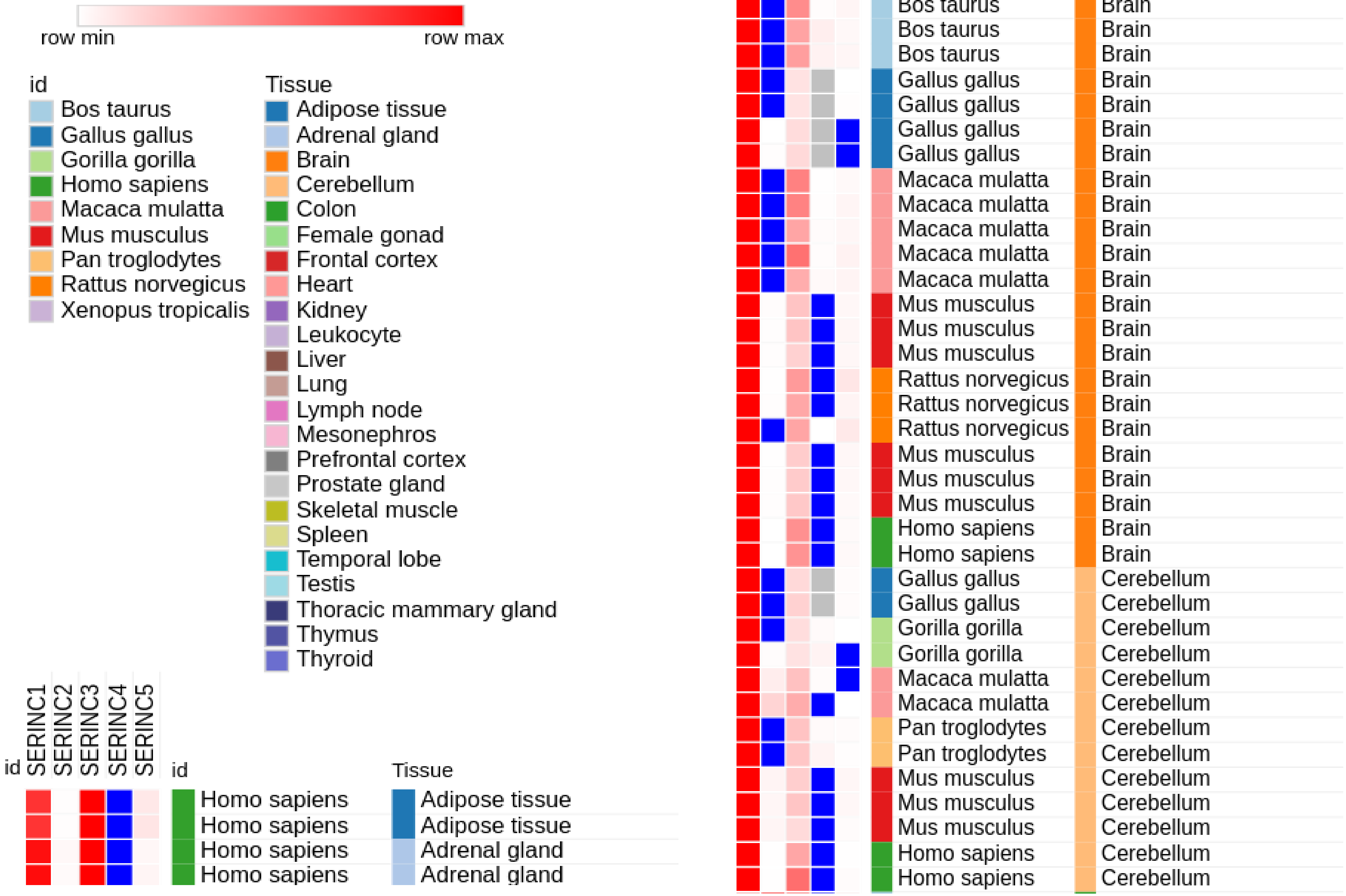

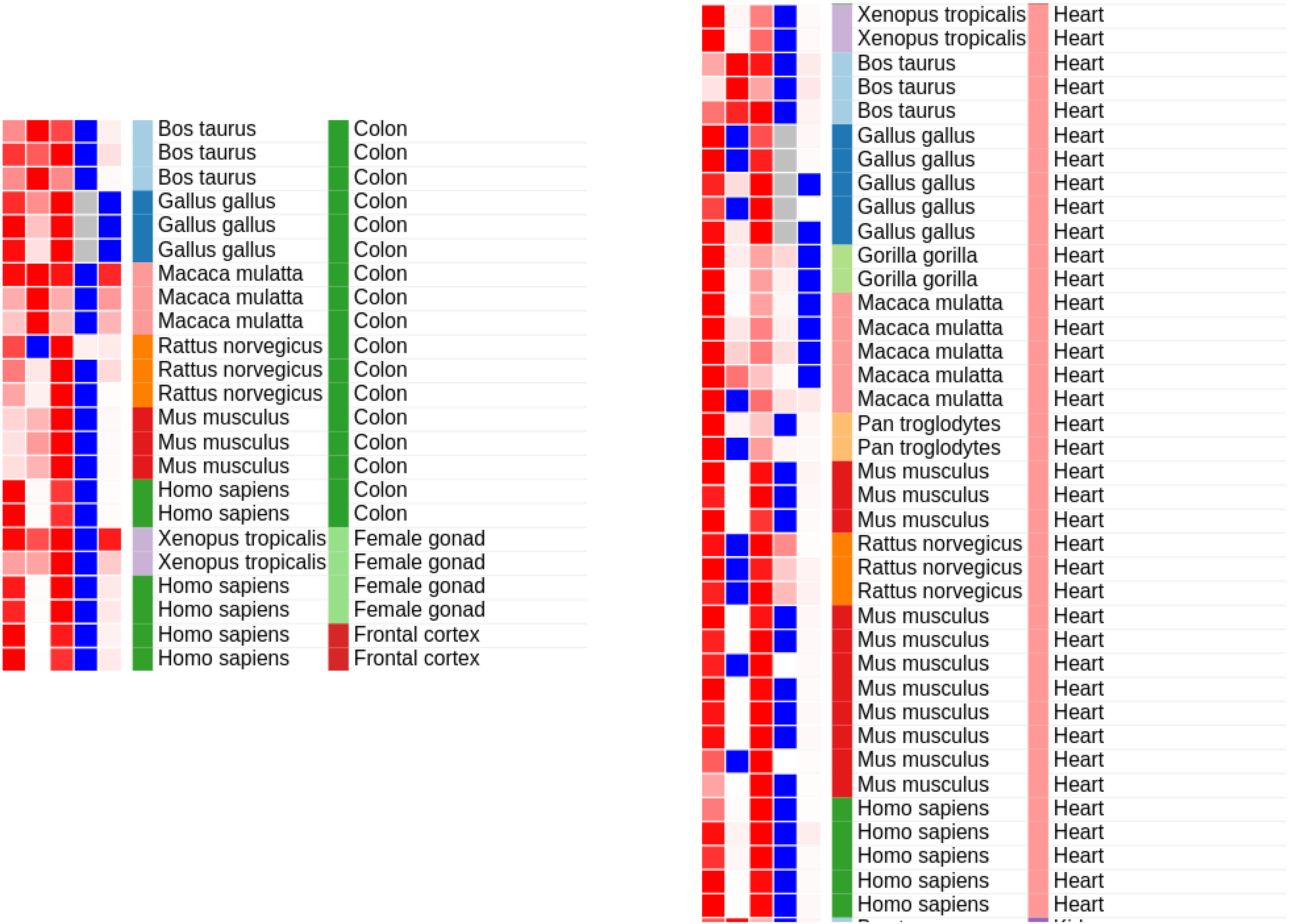
Bos taurus Colon
Bos taurus Colon
Bos taurus Colon
Gallus gallus Colon
Gallus gallus Colon
Gallus gallus Colon
Macaca mulatta Colon
Macaca mulatta Colon
Macaca mulatta Colon
Rattus norvegicus Colon
Rattus norvegicus Colon
Rattus norvegicus Colon
Mus musculus Colon
Mus musculus Colon
Mus musculus Colon
Homo sapiens Colon
Homo sapiens Colon
Xenopus tropicalis Female gonad
Xenopus tropicalis Female gonad
Homo sapiens Female gonad
Homo sapiens Female gonad
Homo sapiens Frontal cortex
Homo sapiens Frontal cortex
Xenopus tropicalis Heart
Xenopus tropicalis Heart
Bos taurus Heart
Bos taurus Heart
Bos taurus Heart
Gallus gallus Heart
Gallus gallus Heart
Gallus gallus Heart
Gallus gallus Heart
Gallus gallus Heart
Gorilla gorilla Heart
Gorilla gorilla Heart
Macaca mulatta Heart
Macaca mulatta Heart
Macaca mulatta Heart
Macaca mulatta Heart
Macaca mulatta Heart
Pan troglodytes Heart
Pan troglodytes Heart
Mus musculus Heart
Mus musculus Heart
Mus musculus Heart
Rattus norvegicus Heart
Rattus norvegicus Heart
Rattus norvegicus Heart
Mus musculus Heart
Mus musculus Heart
Mus musculus Heart
Mus musculus Heart
Mus musculus Heart
Mus musculus Heart
Mus musculus Heart
Mus musculus Heart
Homo sapiens Heart
Homo sapiens Heart
Homo sapiens Heart
Homo sapiens Heart
Homo sapiens Heart

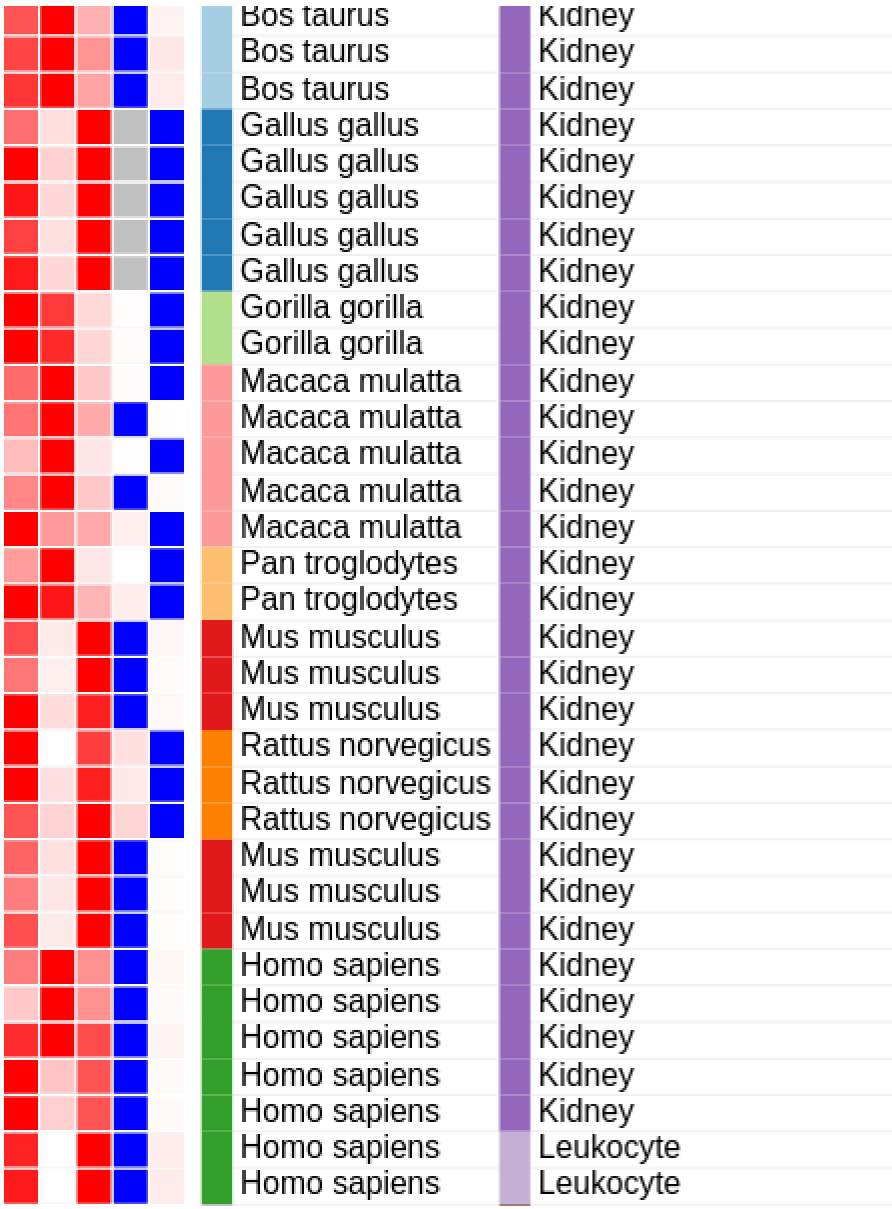

Bos taurus
Kidney
Bos taurus
Kidney
Bos taurus
Kidney
Gallus gallus
Kidney
Gallus gallus
Kidney
Gallus gallus
Kidney
Gallus gallus
Kidney
Gallus gallus
Kidney
Gorilla gorilla
Kidney
Gorilla gorilla
Kidney
Macaca mulatta
Kidney
Macaca mulatta
Kidney
Macaca mulatta
Kidney
Macaca mulatta
Kidney
Macaca mulatta
Kidney
Pan troglodytes
Kidney
Pan troglodytes
Kidney
Mus musculus
Kidney
Mus musculus
Kidney
Mus musculus
Kidney
Rattus norvegicus
Kidney
Rattus norvegicus
Kidney
Rattus norvegicus
Kidney
Mus musculus
Kidney
Mus musculus
Kidney
Mus musculus
Kidney
Homo sapiens
Kidney
Homo sapiens
Kidney
Homo sapiens
Kidney
Homo sapiens
Kidney
Homo sapiens
Kidney
Homo sapiens
Leukocyte
Homo sapiens
Leukocyte

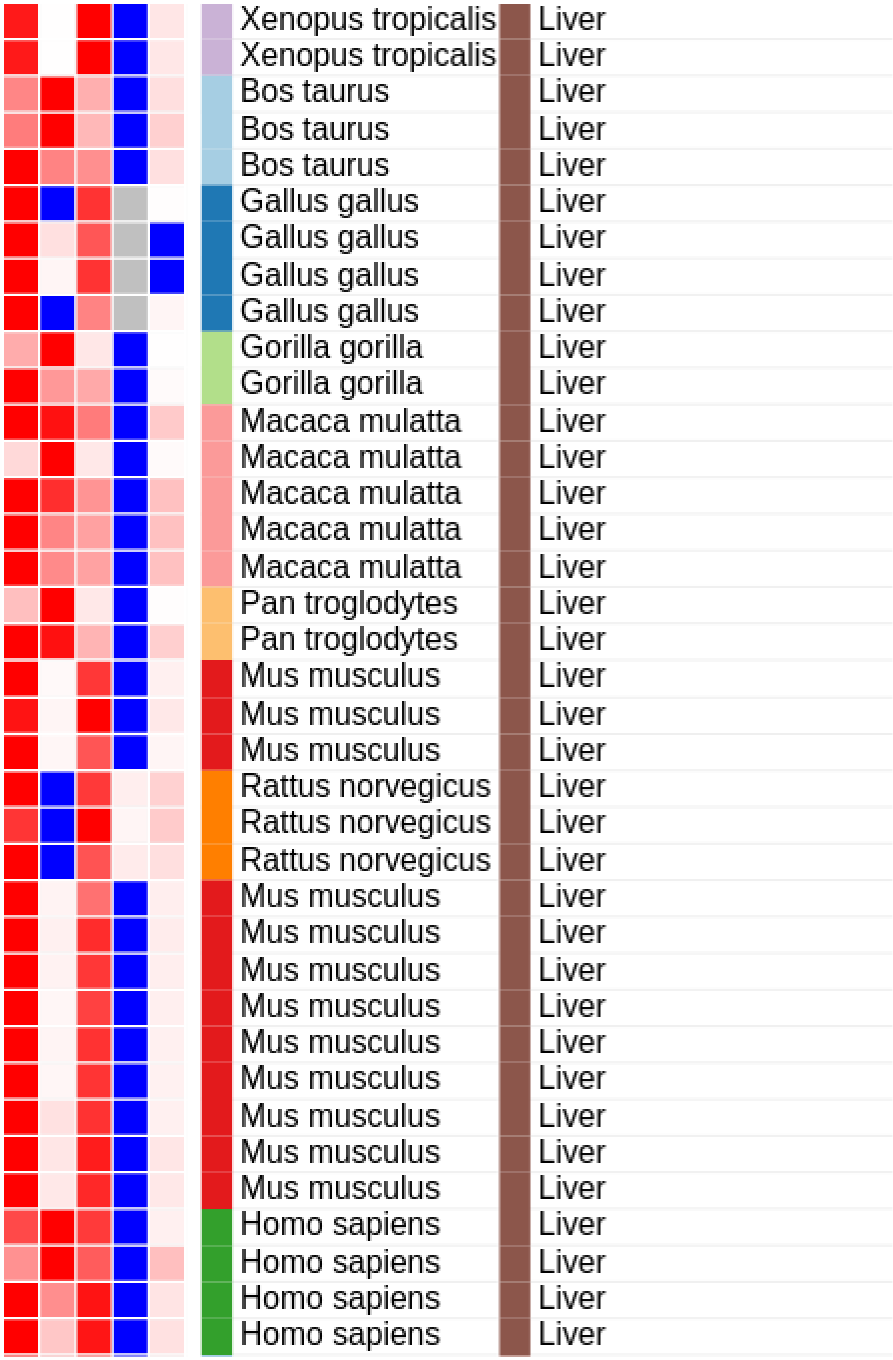

Xenopus tropicalis
Liver
Xenopus tropicalis
Liver
Bos taurus
Liver
Bos taurus
Liver
Bos taurus
Liver
Gallus gallus
Liver
Gallus gallus
Liver
Gallus gallus
Liver
Gallus gallus
Liver
Gorilla gorilla
Liver
Gorilla gorilla
Liver
Macaca mulatta
Liver
Macaca mulatta
Liver
Macaca mulatta
Liver
Macaca mulatta
Liver
Macaca mulatta
Liver
Pan troglodytes
Liver
Pan troglodytes
Liver
Mus musculus
Liver
Mus musculus
Liver
Mus musculus
Liver
Rattus norvegicus
Liver
Rattus norvegicus
Liver
Rattus norvegicus
Liver
Mus musculus
Liver
Mus musculus
Liver
Mus musculus
Liver
Mus musculus
Liver
Mus musculus
Liver
Mus musculus
Liver
Mus musculus
Liver
Mus musculus
Liver
Mus musculus
Liver
Homo sapiens
Liver
Homo sapiens
Liver
Homo sapiens
Liver
Homo sapiens
Liver

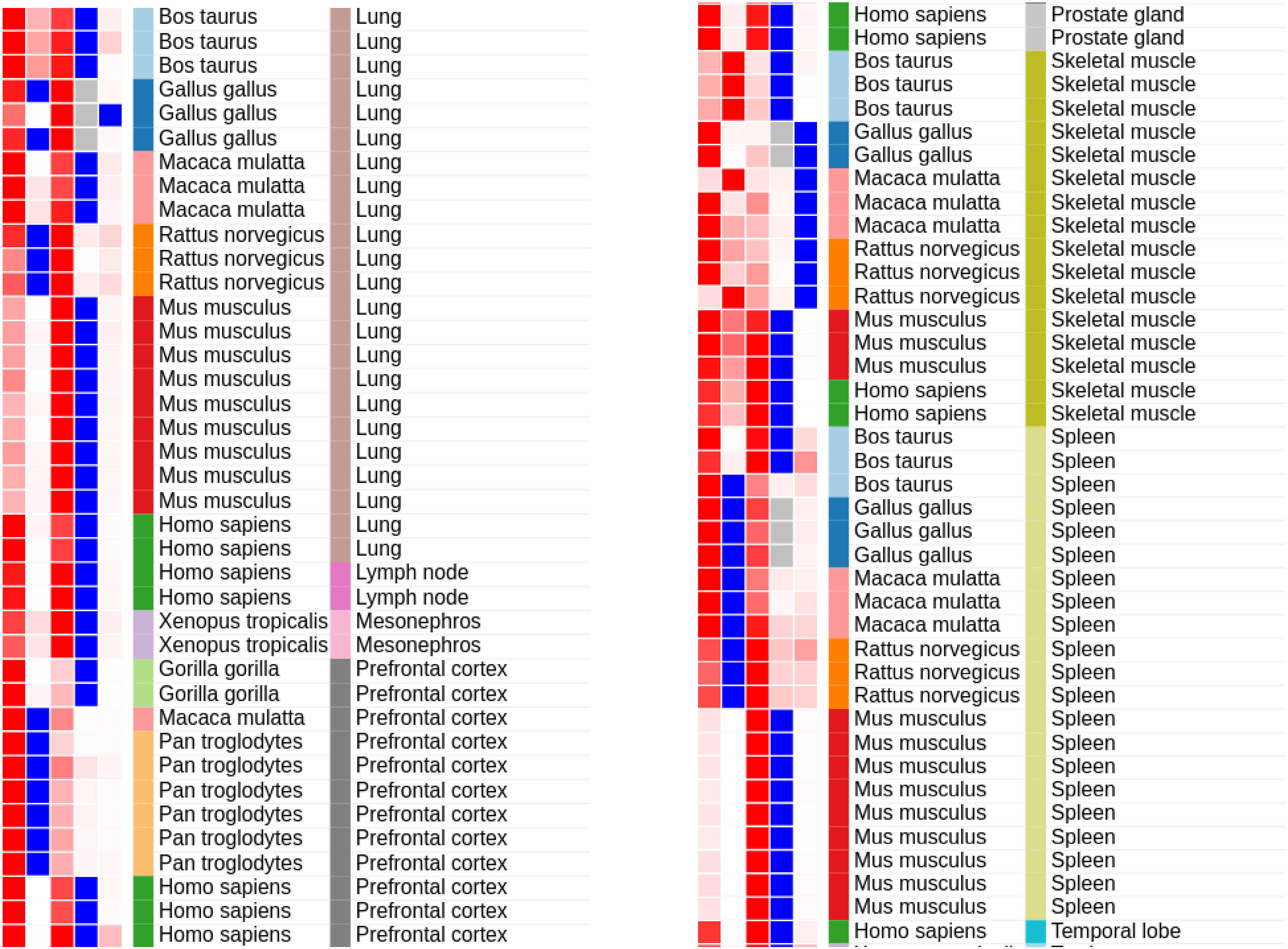

Bos taurus Lung
Bos taurus Lung
Bos taurus Lung
Gallus gallus Lung
Gallus gallus Lung
Gallus gallus Lung
Macaca mulatta Lung
Macaca mulatta Lung
Macaca mulatta Lung
Rattus norvegicus Lung
Rattus norvegicus Lung
Rattus norvegicus Lung
Mus musculus Lung
Mus musculus Lung
Mus musculus Lung
Mus musculus Lung
Mus musculus Lung
Mus musculus Lung
Mus musculus Lung
Mus musculus Lung
Mus musculus Lung
Homo sapiens Lung
Homo sapiens Lung
Homo sapiens Lymph node
Homo sapiens Lymph node
Xenopus tropicalis Mesonephros
Xenopus tropicalis Mesonephros
Gorilla gorilla Prefrontal cortex
Gorilla gorilla Prefrontal cortex
Macaca mulatta Prefrontal cortex
Pan troglodytes Prefrontal cortex
Pan troglodytes Prefrontal cortex
Pan troglodytes Prefrontal cortex
Pan troglodytes Prefrontal cortex
Pan troglodytes Prefrontal cortex
Pan troglodytes Prefrontal cortex
Homo sapiens Prefrontal cortex
Homo sapiens Prefrontal cortex
Homo sapiens Prefrontal cortex
Homo sapiens Prostate gland
Homo sapiens Prostate gland
Bos taurus Skeletal muscle
Bos taurus Skeletal muscle
Bos taurus Skeletal muscle
Gallus gallus Skeletal muscle
Gallus gallus Skeletal muscle
Macaca mulatta Skeletal muscle
Macaca mulatta Skeletal muscle
Macaca mulatta Skeletal muscle
Rattus norvegicus Skeletal muscle
Rattus norvegicus Skeletal muscle
Rattus norvegicus Skeletal muscle
Mus musculus Skeletal muscle
Mus musculus Skeletal muscle
Mus musculus Skeletal muscle
Homo sapiens Skeletal muscle
Homo sapiens Skeletal muscle
Bos taurus Spleen
Bos taurus Spleen
Bos taurus Spleen
Gallus gallus Spleen
Gallus gallus Spleen
Gallus gallus Spleen
Macaca mulatta Spleen
Macaca mulatta Spleen
Macaca mulatta Spleen
Rattus norvegicus Spleen
Rattus norvegicus Spleen
Rattus norvegicus Spleen
Mus musculus Spleen
Mus musculus Spleen
Mus musculus Spleen
Mus musculus Spleen
Mus musculus Spleen
Mus musculus Spleen
Mus musculus Spleen
Mus musculus Spleen
Mus musculus Spleen
Homo sapiens Temporal lobe

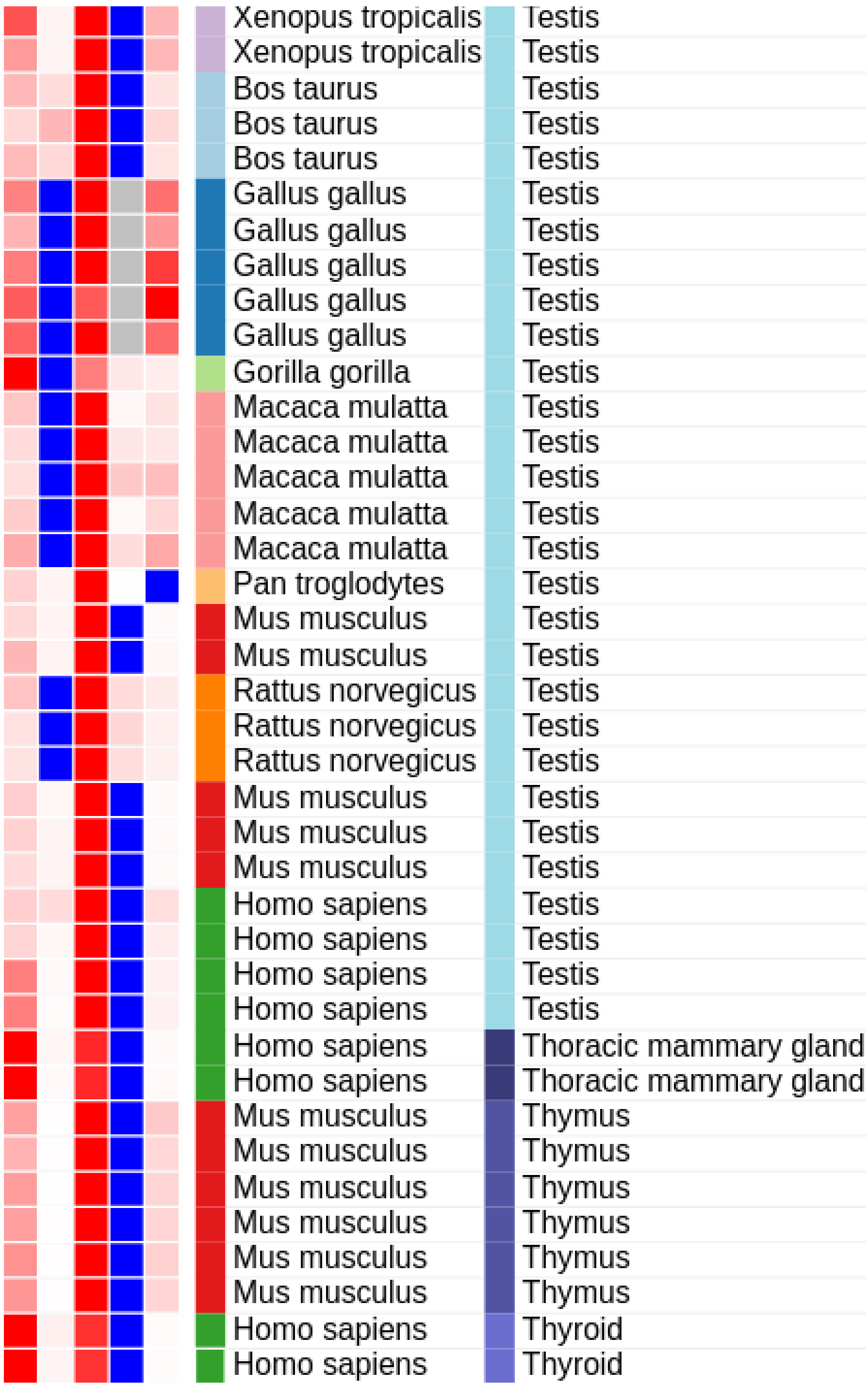
Xenopus tropicalis
Testis
Xenopus tropicalis
Testis
Bos taurus
Testis
Bos taurus
Testis
Bos taurus
Testis
Gallus gallus
Testis
Gallus gallus
Testis
Gallus gallus
Testis
Gallus gallus
Testis
Gallus gallus
Testis
Gorilla gorilla
Testis
Macaca mulatta
Testis
Macaca mulatta
Testis
Macaca mulatta
Testis
Macaca mulatta
Testis
Macaca mulatta
Testis
Pan troglodytes
Testis
Mus musculus
Testis
Mus musculus
Testis
Rattus norvegicus
Testis
Rattus norvegicus
Testis
Rattus norvegicus
Testis
Mus musculus
Testis
Mus musculus
Testis
Mus musculus
Testis
Homo sapiens
Testis
Homo sapiens
Testis
Homo sapiens
Testis
Homo sapiens
Testis
Homo sapiens
Thoracic mammary gland
Homo sapiens
Thoracic mammary gland
Mus musculus
Thymus
Mus musculus
Thymus
Mus musculus
Thymus
Mus musculus
Thymus
Mus musculus
Thymus
Mus musculus
Thymus
Homo sapiens
Thyroid
Homo sapiens
Thyroid
